# Inconsistencies in the Classification of the Family Cydnidae (Hemiptera: Heteroptera: Pentatomoidea) Revealed by Molecular Apomorphies in the Secondary and Tertiary Structures of 18S rRNA Length-Variable Region L (LVR L)

**DOI:** 10.3390/ijms25020939

**Published:** 2024-01-11

**Authors:** Jerzy A. Lis, Paweł J. Domagała

**Affiliations:** Institute of Biology, University of Opole, Oleska 22, 45-052 Opole, Poland; pdomagala@uni.opole.pl

**Keywords:** *Cydnidae*, cydnoid complex, phylogeny, classification, molecular apomorphies

## Abstract

The SSU nuclear rDNA (encoding 18S ribosomal RNA) is one of the most frequently sequenced genes in the molecular analysis of insects. Molecular apomorphies in the secondary and tertiary structures of several 18S rRNA length-variable regions (LVRs) located within the V2, V4, and V7 hypervariable regions can be good indicators for recovering monophyletic groups within some heteropteran families. Among the LVRs that have been analysed, the LVR L in the V4 hypervariable region is the longest and most crucial for such assessments. We analysed the 18S rRNA V4 hypervariable region sequences of 45 species from the family Cydnidae, including all 6 subfamilies (Amaurocorinae, Amnestinae, Cephalocteinae, Cydninae, Garsauriinae, and Sehirinae) and three pentatomoid families (Parastrachiidae, Thaumastellidae, and Thyreocoridae), which have often been included in the broadly defined Cydnidae family. This is the first time that representatives of all Cydnidae subfamilies have been included in a molecular analysis. Only taxa from two subfamilies, Sehirinae and Cydninae, have been used in previous molecular studies. The secondary and tertiary structures of the LVR L were predicted for each species using the two-step procedure already accepted for such analyses to recover any molecular apomorphy essential for determining monophyly. The results of our comparative studies contradict the current understanding of the relationships among burrowing bugs and the current family classification.

## 1. Introduction

Recent studies [1,2] have demonstrated that, in addition to the 18S rDNA sequence analyses that are commonly employed to establish phylogenetic relationships among taxa within Heteroptera, e.g., [3,4,5,6,7,8,9,10,11,12,13], investigations comparing the secondary and tertiary structures of ribosomal RNA encoded by this gene are also important.

Both studies [1,2] confirmed previous suggestions [5,14,15,16,17] that three hypervariable regions (V2, V4, and V7) containing numerous length-variable regions (LVRs) are critical for such analyses. Certain LVRs secondary and tertiary structures may act as apomorphies for monophyletic groups that have been recovered during the phylogenetic analysis of Heteroptera [1,2,15,16,17]. Of the thirteen LVRs located within the V2, V4, and V7 hypervariable regions of the 18S rRNA [15,16,17], the LVR L in the V4 region contains secondary and tertiary structures that can serve as morpho-molecular synapomorphies or autapomorphies for monophyletic groups within this hemipteran suborder [1,2].

One study [1] focused on the Thaumastellidae species of the superfamily Pentatomoidea; therefore, we aimed to confirm if analysing the LVR L’s secondary and tertiary structures in the closely related family Cydnidae would reveal the morpho-molecular apomorphies necessary to define the monophyletic groups within the family. We chose Cydnidae because it is one of the least-studied Pentatomoidea families, and its classification remains controversial [1,4,10,11,12,18,19,20,21,22,23,24,25,26,27,28].

There are two different subfamily classifications of Cydnidae currently used. In the broadest sense [18], the family Cydnidae includes all taxa that are morphologically defined by the presence of a series of flattened setae forming coxal combs not found elsewhere among Heteroptera [20]. One argument is that the family contains nine subfamilies: Amaurocorinae, Amnestinae, Cephalocteinae, Cydninae, Garsauriinae, Parastrachiinae, Sehirinae, Taumastellinae, and Thyreocorinae. However, the characteristic morphological feature described has independently evolved several times within the family [4]; therefore, this group cannot be clearly defined, and the family classification has not been accepted.

The other classification recognises only six subfamilies within Cydnidae: Amnestinae, Amaurocorinae, Cephalocteinae (combining Cephalocteini and Scaptocorini), Cydninae (combining Cydnini and Geotomini *sensu lato*), Garsauriinae, and Sehirinae *sensu lato*. This classification is currently sanctioned and widely accepted [1,9,10,19,21,25,27,29,30,31,32,33], and was considered the baseline for our analyses. In this definition, three taxa, Parastrachiidae, Thaumastellidae and Thyreocoridae (combining Thyreocorinae and Corimelaeninae), are considered separate Pentatomoidea families.

The present study aimed to verify whether the secondary and tertiary structures of the 18S rRNA LVR Ls of representatives of the six Cydnidae subfamilies demonstrate morpho-molecular apomorphies that could serve as indicators of monophyly for certain taxa groups.

## 2. Results

### 2.1. Hypervariable Region V4 and Length-Variable Region L (LVR L) Sequence Analyses

**Figure A1:**
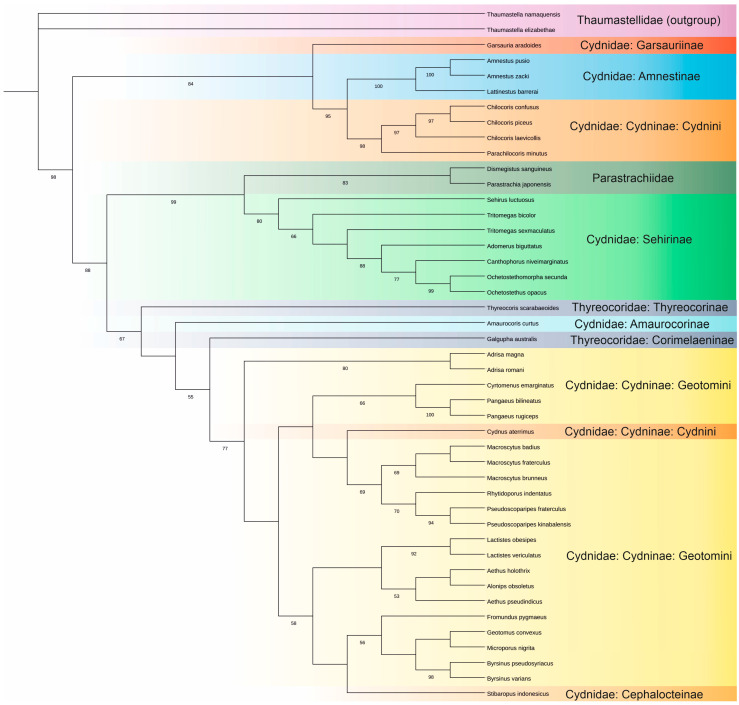
Phylogenetic consensus tree of the family Cydnidae based on the Maximum Likelihood analysis of the sequences of the *18S rDNA* hypervariable region V4. Node labels—Ultrafast Bootstrap values (see Section 4.5), and the Bootstrap values over 50% are shown next to the branches.

The 18S rDNA sequences in the V4 hypervariable regions of 45 Pentatomoidea species were phylogenetically analysed to identify monophyletic groups (Figure A1) and their ‘consensus species’, which are crucial for predicting the secondary and tertiary structures of the LVR L during further analyses [1,2]. Thirty sequences were newly acquired and deposited in GenBank. Their accession numbers are listed in Tables S3 and S4.

The examined taxa included 2 species in the outgroup (Thaumastellidae) and 43 species in the ingroup (two species of the Parastrachiidae, 2 species of the Thyreocoridae, and 39 species of the Cydnidae). The latter family included members of all its six currently recognised subfamilies [19,20,21], namely Amaurocorinae (one species), Amnestinae (three species), Cephalocteinae (one species), Cydninae (twenty-six species), Garsauriinae (one species), and Sehirinae (seven species) (Table S1).

The examined taxa included 2 outgroup species (Thaumastellidae) and 43 ingroup species (2 species from Parastrachiidae, 2 from Thyreocoridae, and 39 from Cydnidae). All six currently recognised Cydnidae subfamilies [19,20,21] were included: Amaurocorinae (one species), Amnestinae (three species), Cephalocteinae (one species), Cydninae (twenty-six species), Garsauriinae (one species), and Sehirinae (seven species) (Table S1).

For the first time, the family Cydnidae was represented by species belonging to all subfamilies and almost all tribes. One tribe, the Cephalocteini, was not represented in the present analysis.

To verify the sequence variability within the tribes identified as polyphyletic by Pluot-Sigwalt and Lis [19], such as Geotomini *sensu lato* (subfamily Cydninae) and Sehirini *sensu lato* (subfamily Sehirinae), all analysed species were assigned to the corresponding spermathecal types, and facies recovered within these tribes [19].

The final 18S rDNA V4 hypervariable region alignment contained 331 sites. There were 219 and 112 conserved and variable sites, respectively, while 78 were parsimony-informative and 34 were singletons. The alignment file used to search for monophyletic groups is available in the Supplementary Material (File S1).

ModelFinder in the IQ-TREE [34] tested 88 DNA models for this set of sequences. The K3P + G4 substitution model was selected as the best fit based on the Bayesian Information Criterion. The IQ-TREE generated 98 initial trees; the ML consensus tree is shown in Figure A1.

The number of nucleotides in the hypervariable region V4, the LVR L, and the L2 subregion of LVR L in the 18S rRNA of the ‘consensus species’ for families, subfamilies, facies, and clades within tribes (Figure A1) are given in Table 1. The same data for every species analysed are provided in Table S1.

The number of nucleotides for the higher taxa (families, subfamilies, and tribes) for each region or subregion is shown in Table 2. The same data for all the species are provided in Table S2.

The length of the analysed hypervariable region V4 varies significantly within the family Cydnidae (316–325 nucleotides), while it shows less variation in other ‘cydnoid’ families—specifically 316 nucleotides in both Parastrachiidae and Thyreocoridae, and 315 and 318 in Thaumastellidae (Table 1, Table 2 and Table S1).

The length of the hypervariable region V4 varied significantly within the family Cydnidae (316–325 nucleotides), while less variation was observed in other ‘cydnoid’ families. The length was 316 nucleotides in both Parastrachiidae and Thyreocoridae and 315 and 318 in Thaumastellidae (Table 1, Table 2 and Table S1).

For the family Cydnidae, the nucleotide number in this region is most consistent within the geotoman facies of the tribe Geotomini *s. lato*, in which all species have 317 nucleotides (Table S1). Only one species within the subfamily Cephalocteinae, which also has the geotoman facies of the spermatheca, and one representative of the subfamily Garsauriinae, which has the garsauriinae type of spermatheca, shared the same nucleotide number (i.e., 317) (Table S1).

The shortest length of the hypervariable region V4 within the family Cydnidae, namely 316, characterises all species representing the subfamily Sehirinae (Table S1). These 316 nucleotides were found in several Cydnidae taxa and in representatives of the families Thyreocoridae and Parastrachiidae and the subfamily Sehirinae. These include the only member of the subfamily Amaurocorinae (characterised by the amaurocorinae type of spermatheca) and five members of Geotomini *s. lato* tribe (subfamily Cydninae). The latter includes two adrisan facies species (*Adrisa magna* and *A. romani*), two scoparipan facies species (*Pseudoscoparipes fraterculus* and *P. kinabalensis*), and one cydnan facies species (*Cydnus aterrimus*) (Table S1).

The highest number of nucleotides in the hypervariable region V4 was found in the subfamily Amnestinae (324 nucleotides) and in most species representing the tribe Cydnini (three species of *Chilocoris* and one of *Parachilocoris*) (325 nucleotides). These are the highest nucleotide numbers recovered in the hypervariable region V4 among all taxa of the superfamily Pentatomoidea [1]. Such high nucleotide numbers are unique to Cydnidae and the entire superfamily Pentatomoidea [1].

The high number of nucleotides in the *Chilocoris* and *Parachilocoris* species (representing the tribe Cydnini) correlated with a significantly higher number of nucleotides in the LVR L region (81 nucleotides). However, the high number of nucleotides found in the hypervariable region V4 in species of the subfamily Amnestinae did not correlate with a higher number of nucleotides in their LVR L region. All Amnestinae species had 73 nucleotides in the LVR L region. This is the same as in many other species of the subfamily Cydninae (adrisan and scoparipan facies), all of the Sehirinae, and all of the family Parastrachiidae (Table 1, Table 2 and Table S1). Seventy-four nucleotides within the LVR L region (Table 1, Table 2 and Table S1) were observed among species of the subfamily Garsauriinae, the Cephalocteinae, all geotoman facies of the subfamily Cydninae, and species of the family Thyreocoridae.

The number of nucleotides within the L2 subregion of the LVR L varied from 3–7 (Table 1 and Table 2, Tables S1 and S2). This region is discussed in detail within the context of the secondary structure of LVR L (see Section 2.3).

### 2.2. 18S rRNA Secondary and Tertiary Structure Models

**Figure 1:**
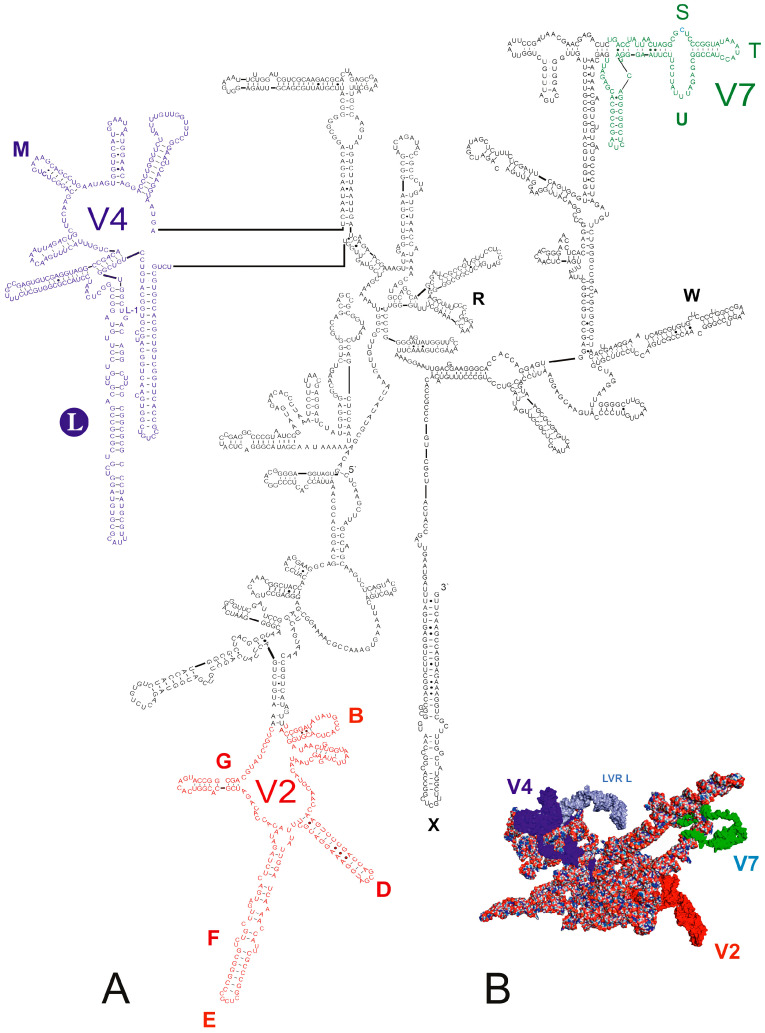
*18S rRNA* of *Fromundus pygmaeus* (**A**) Secondary structure model. The bases marked in colour represent the hypervariable regions (V2—red, V4—dark blue, V7—green). Thirteen length-variable regions (LVRs) are labelled as capital letters B to W in colours analogous to the base colours representing the hypervariable regions or other sequences’ regions. The capital letter in the filled circle indicates the LVR L. Base pairing is shown as follows: standard canonical pairs are lines (G–C, A–U), wobble G:U pairs are dots (G·U), A:G or A:C pairs are open circles (A; G, A; C) and other non-canonical pairs are filled circles (e.g., U and U, A and A). (**B**) Tertiary structure model. The fragments marked in colour represent the hypervariable regions (V2—red, V4—dark blue, V7—green). The LVR L region within the V4 hypervariable region is marked in dark grey.

The secondary and tertiary structure models of the predicted 18S rRNA gene for *Fromundus pygmaeus* (subfamily Cydninae) and *Adomerus biguttatus* (subfamily Sehirinae) are shown in Figure 1 and Figure S1, respectively. For both predictions, existing 18S rDNA sequences deposited in GenBank (*F. pygmaeus*, GenBank accession number KJ535871; *A. biguttatus*, GenBank accession number KY886253) were used.

Both models were generally consistent with those for all pentatomoid species analysed to date [1,2,15,16,17], particularly when considering the location of three hypervariable regions (V2, V4, V7) and the position of the LVR L within the gene sequences (Figure 1 and Figure S1).

### 2.3. Length-Variable Region L Secondary Structure

The LVR L length among the observed species ranged from 72–81 nucleotides and exhibited the most notable variation in the subfamily Cydninae of the Cydnidae (73–81 nucleotides), as shown in Table 2 and Table S1.

**Figure 2:**
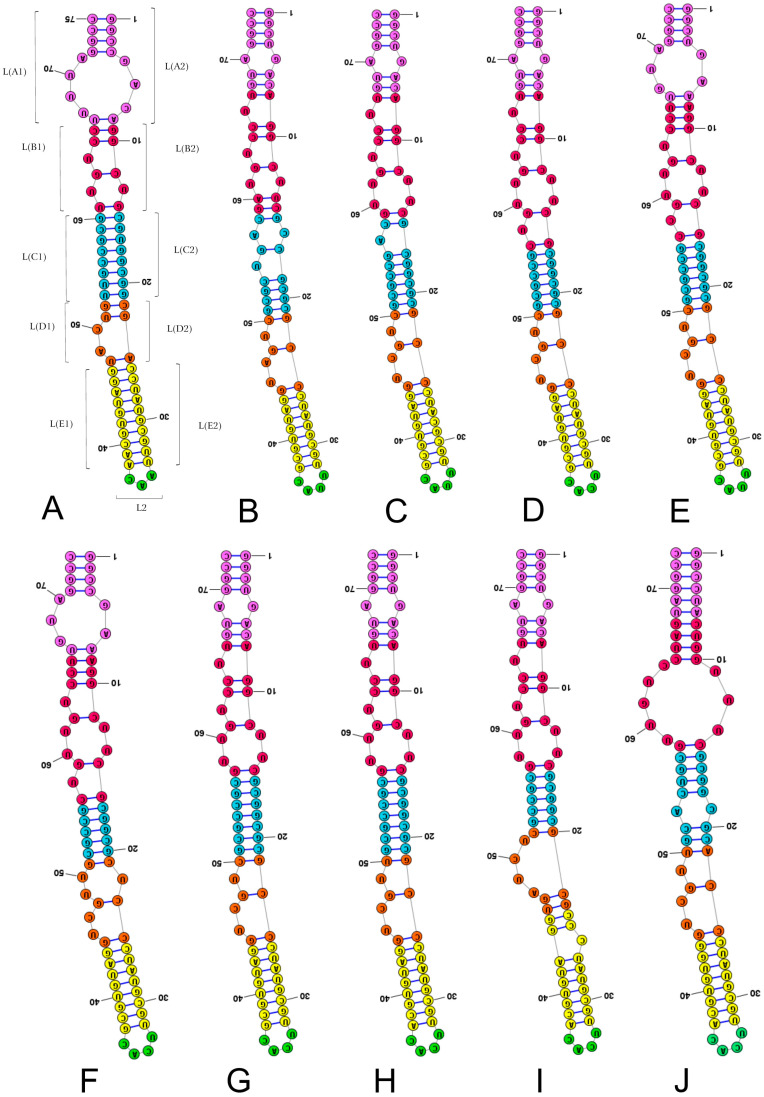
Secondary structure models of the length-variable region L characterised by 3–4 nucleotides in the subregion L2. (**A**) *Thaumastella elizabethae* (Thaumastellidae, outgroup). (**B**) *Stibaropus indonesicus* (Cydnidae: Cephalocteinae). (**C**) *Fromundus pygmaeus* (Cydnidae: Cydnidae: Geotomini). (**D**) *Macroscytus badius* (Cydnidae: Cydnidae: Geotomini). (**E**) *Cyrtomenus emarginatus* (Cydnidae: Cydnidae: Geotomini). (**F**) *Rhytidoporus indentatus* (Cydnidae: Cydnidae: Geotomini). (**G**) *Adrisa romani* (Cydnidae: Cydnidae: Geotomini). (**H**) *Pseudoscoparipes fraterculus* (Cydnidae: Cydnidae: Geotomini). (**I**) *Cydnus aterrimus* (Cydnidae: Cydnidae: Cydnini). (**J**) *Ochetostethomorpha secunda* (Cydnidae: Sehirinae: Sehirini). Specific subregion bases are marked in the same colour.

**Figure 3:**
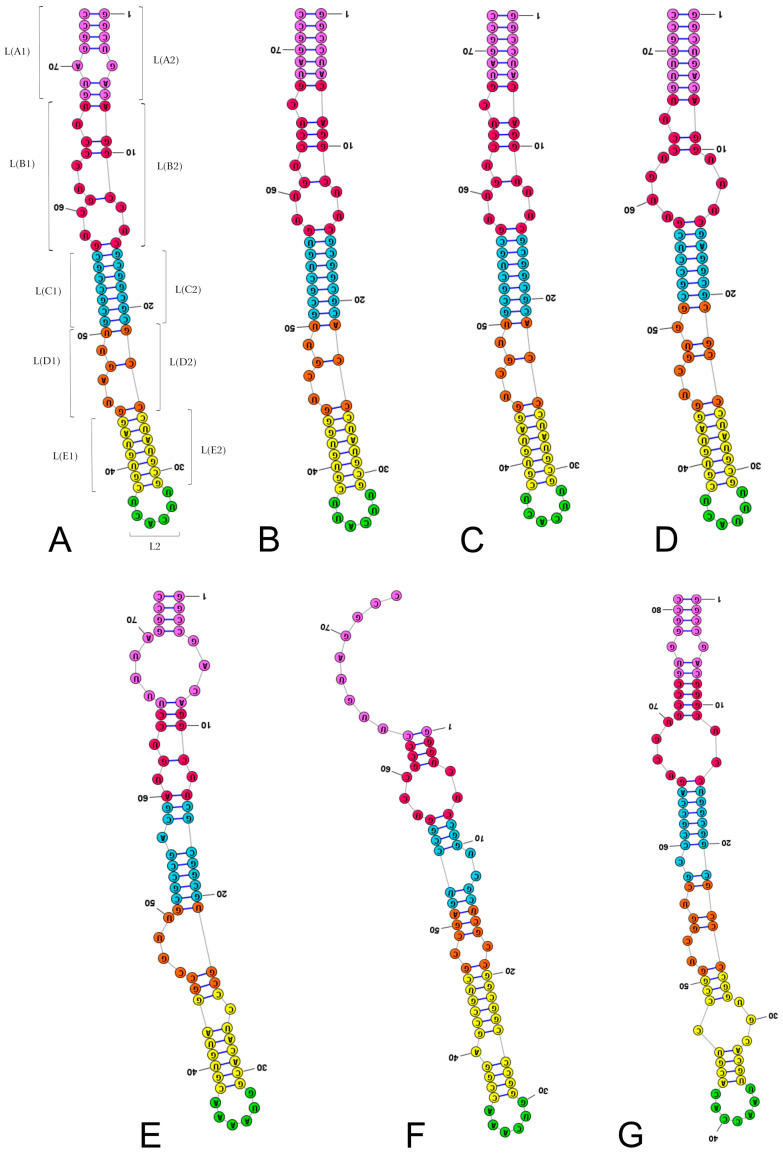
Secondary structure models of the length-variable region L characterised by 6–7 nucleotides in the subregion L2. (**A**) *Thyreocoris scarabaeoides* (Thyreocoridae). (**B**) *Parastrachia japonensis* (Parastrachiidae). (**C**) *Adomerus buguttatus* (Cydnidae: Sehirinae: Sehirini). (**D**) *Amaurocoris curtus* (Cydnidae: Amaurocorinae). (**E**) *Garsauria aradoides* (Cydnidae: Garsauriinae). (**F**) *Amnestus zacki* (Cydnidae: Amnestinae). (**G**) *Chilocoris piceus* (Cydnidae: Cydninae: Cydnini). Specific subregion bases are marked in the same colour.

Subregions were shaped to identify homologous fragments in the LVR L sequences of the investigated species (Table 3, Figure 2, Figure 3 and Figure S2) following recent analyses of the 18S rRNA secondary structures in the superfamily Pentatomoidea [1]. The subdivision of LVR L into subregions was based on the alignment (File S1, Figure S2) and results of the secondary structure predictions (Figure 2 and Figure 3). The number of nucleotides for each subregion based on this comparative analysis is provided in Table 3.

LA and LB were the most variable subregions in terms of nucleotide numbers, ranging from 11–17 and 11–19, respectively (Table 3). All other subregions showed significantly less variation, with nucleotide numbers ranging from 3–7 in L2, 12–16 in LC, 8–11 in LD, and 16–21 in LE (Table 3). LD was the most consistent subregion across species, with ten species containing the same nucleotide formula (6 + 3) (Table 3).

Only two species had the same number of nucleotides in each of the subregions analysed: *Adrisa romani* and *Pseudoscoparipes fraterculus*, representing the tribe Geotomini of the subfamily Cydninae. Most importantly, the consensus species of the subfamily Amnestinae demonstrated autapomorphic nucleotide numbers in all the subregions except LD and L2. The other ingroup taxa demonstrated single autapomorphies (Thyreocoridae in the subregion LB, *Cydnus aterrimus* in the subregion LE) or two autapomorphies (*Chilocoris piceus* in the subregions L2 and LE). The outgroup consensus species representing Thaumastellidae showed two autapomorphies in the L2 and LE subregions (Table 3).

Comparing the secondary structures of each subregion (File S2), the LB subregion was found to be the most variable in nucleotide numbers, with 11 different secondary structures observed. Despite its high variability in nucleotide numbers (11–17), the LA subregion only formed six different secondary structures (File S2). The LC subregion, which showed slight variation in nucleotide number (12–16), demonstrated 10 different secondary structures (File S2). Two other subregions (LD, LE) formed six and seven secondary structures, respectively (File S2).

Most species (denoted by the number 4 in File S2) had specific secondary structure of the LA subregion. This group of eight species come from the Cydnidae (three from the Cydninae and one from the Cephalocteinae), while one species is from the Thyreocoridae (see File S2). Both species of the subfamily Sehirinae (*O. secunda*, *A. biguttatus*) had the same secondary structure in the LA subregion as *P. japonensis* (Parastrachiidae) (marked as number 5 in File S2).

The LA subregion showed the most asymmetric secondary structures, with 10 nucleotides in L(A1) and a single nucleotide in L(A2), in the consensus species of the subfamily Amnestinae (*A. zacki*) (Figure S2, Table 3, File S2). All other species showed symmetric structures (Figure S2, Table 3, File S2).

The LB subregion was the most diverse of all the LVR L subregions, with only one structure shared by more than two species. This structure, designated number 6 within the LB subregion in File S2, was recovered in four species: three species of the tribe Geotomini *sensu lato* and one of the tribe Cydnini (all of the subfamily Cydninae of the fa-mily Cydnidae). One or two species represented all other secondary structure types within the LB subregion (File S2).

Although the LC subregion was characterised by low variability in nucleotide number (maximum range of five), 10 different types of secondary structures were noted. The most common was numbered 6 within the LC subregion (File S2). Three species that possessed this structure belonged to the Cydnidae (two of the subfamily Cydninae, one of the Sehirinae), one belonged to the family Parastrachiidae, and one belonged to the family Thyreocoridae.

The LD subregion was the most constant fragment of the entire LVR L. Ten species had the same nucleotide numbers and arrangement (6 + 3) (Table 3) and the same secondary structure (numbered 5 in File S2). This group included eight species of the family Cydnidae (a single species of the subfamily Cephalocteinae, five species of the subfamily Cydninae, and two of the subfamily Sehirinae) and one species each of the families Parastrachiidae and Thyreocoridae (File S2).

In the LE subregion, seven different types of secondary structures were distinguished (File S2); however, most species demonstrated only one of two (Table 3, File S2). The first, numbered 5 in File S2, was found in seven species of the family Cydnidae (one of the Cephalocteinae, five of the Cydninae, and one of the Sehirinae) and a single species of the family Parastrachiidae (File S2). The second group (designated 7 in File S2) consisted of one species each from the families Amaurocorinae, Sehirinae, Parastrachiidae, and Thyreocoridae.

The number of nucleotides in the L2 subregion enabled all analysed species to be placed in four groups (Table 3, File S2). Three nucleotides were only found in Thaumastellidae, distinguishing it as the outgroup species of the superfamily. Seven nucleotides were only observed in one species representing the tribe Cydnini (subfamily Cydninae of the Cydnidae). The two remaining L2 groups, characterised by four and six nucleotides, contained many more species (nine and six, respectively). The first group had seven species of Cydninae, one of Cephalocteinae, and one of Sehirinae (Table 3, File S2). The second group contained a single species each from the Parastrachiidae, Thyreocoridae, Amnestinae, Garsauriinae, Amaurocorinae, and Sehirinae (the last four of the family Cydnidae) families (File S2).

### 2.4. Length-Variable Region L Tertiary Structure

**Figure 4:**
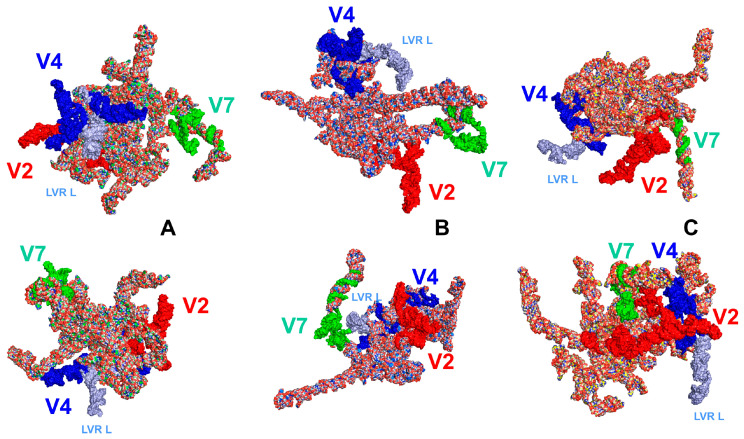
The LVR L position (marked in light blue) within the hypervariable region V4 in the tertiary structure models of the *18S rRNA* gene in two different views. (**A**) *Thaumastella elizabethae* (Thaumastellidae, outgroup [1]). (**B**) *Fromundus pygmaeus* (Cydnidae: Cydninae). (**C**) *Adomerus biguttatus* (Cydnidae: Sehirinae). The hypervariable regions are marked in red (V2), dark blue (V4) and green (V7). All sequences were aligned to the outgroup sequence.

The position of the LVR L in the gene tertiary structure for the outgroup (*T. elizabethae*) and two ingroup species (*F. pygmaeus*, *A. biguttatus*) is shown in Figure 4.

**Figure 5:**
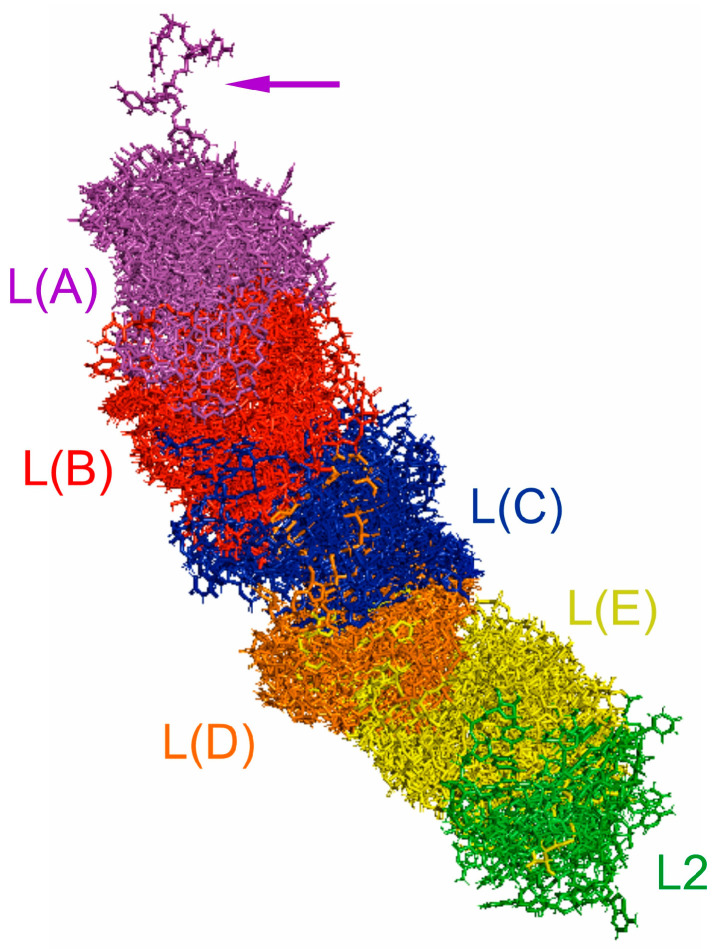
All analysed LVR L tertiary structures divided into subregions and aligned with the outgroup (*Thaumastella elizabethae*) structure. The arrow indicates the LVR L(A) of *Amnestus zacki*. The subregions are marked in colours according to Figure 2 and Figure 3.

When all 16 analysed LVR L tertiary structures were combined and aligned to the outgroup sequence (Figure 5), their general shapes appeared very similar. Only one species, *Amnestus zacki*, had an LVR L tertiary structure fragment (LVR LA) distinct from those found in other species. This could undoubtedly serve as one of its potential morpho-molecular autapomorphies.

Root-mean-square deviation (RMSD) values (calculated using the RNAssess web server [35] and PyMol software v. 2.5.2 [36]) for each LVR L sequence aligned with the outgroup model are shown in Table 4. The results of the analysis of all LVR L tertiary structures for the sequences of the ingroup species, including the calculation of the Interaction Network Fidelity (INF), Deformation Profile (DP), and *p*-value coefficients, are provided in File S3. The results of the RMSD calculations for the specific tertiary structures of the LVR L subregions, which could serve as potential morpho-molecular synapomorphies for two species, are shown in Table 5. The RMSD calculation results for the LVR L subregions that could serve as morpho-molecular synapomorphies for more than two species are shown in Table 6. The RMSD values for the LVR L subregions that can be considered reliable morpho-molecular autapomorphies are shown in Table 7.

**Table 4:** RMSD values (calculated in RNAssess web server and PyMol software) v. 2.5.2 for each LVR L sequence model aligned to the outgroup (*Thaumastella elizabethae*, Thaumastellidae) model.

Model	Family, Subfamily, Tribe	RMSD Value	
		RNAssess Web Server	PyMol
*Rhytidoporus indentatus*	Cydnidae: Cydninae: Geotomini	5.47	5.477
*Cyrtomenus emarginatus*	Cydnidae: Cydninae: Geotomini	5.88	5.881
*Thyreocoris scarabaeoides*	Thyreocoridae: Thyreocorinae	6.28	6.277
*Macroscytus badius*	Cydnidae: Cydninae: Geotomini	6.46	6.459
*Stibaropus indonesicus*	Cydnidae: Cephalocteinae: Scaptocorini	6.49	6.483
*Fromundus pygmaeus*	Cydnidae: Cydninae: Geotomini	6.58	6.584
*Pseudoscoparipes fraterculus*	Cydnidae: Cydninae: Geotomini	6.70	6.693
*Adomerus biguttatu*	Cydnidae: Sehirinae: Sehirini	6.82	6.824
*Garsauria aradoides*	Cydnidae: Garsauriinae	6.85	6.851
*Parastrachia japonensis*	Parastrachiidae	7.17	7.170
*Adrisa romani*	Cydnidae: Cydninae: Geotomini	7.73	7.725
*Cydnus aterrimus*	Cydnidae: Cydninae: Cydnini	8.47	8.468
*Ochetostethomorpha secunda*	Cydnidae: Sehirinae: Sehirini	9.81	9.811
*Amaurocoris curtus*	Cydnidae: Amaurocorinae	11.33	11.332
*Chilocoris piceus*	Cydnidae: Cydninae: Cydnini	11.73	11.733
*Amnestus zacki*	Cydnidae: Amnestinae	15.12	15.116

**Table 5:** RMSD values (calculated in RNAssess web server and PyMol) for the specific tertiary structure of the LVR L subregions, which can serve as potential morpho-molecular synapomorphies for two species analysed (target and compared models). The value from the calculation containing more atoms was deemed conclusive. Figure 6 and Figure 7 were used as the basis for the numbering of synapomorphies.

Subregion Compared [Synapomorphy Numbering as in Figure 6 and Figure 7]	Target Model	Compared Model	RMSD Value	
			RNAssess Web Server (Number of Atoms Compared)	PyMol (Number of Atoms Compared)
L(A) [s1] L(A) [s2]	*Thaumastella elizabethae*	*Garsauria aradoides*	3.98 (413)	0.194 (543)
	*Adomerus biguttatus*	*Parastrachia japonensis*	0.20 (352)	0.201 (352)
L(B) [s3]	*Cyrtomenus emarginatus*	*Rhytidoporus indentatus*	0.21 (538)	0.205 (556)
L(C) [s4]	*Adomerus biguttatus*	*Parastrachia japonensis*	0.25 (422)	0.252 (423)
L(C) [s5]	*Macroscytus badius*	*Cyrtomenus emarginatus*	0.10 (390)	0.104 (390)

**Table 6:** RMSD values (calculated in RNAssess web server and PyMol) for the specific tertiary structure of the LVR L subregions, which can serve as potential morpho-molecular synapomorphies for more than two analysed species (one target and several compared models). The value from the calculation containing more atoms was deemed conclusive.

Subregion Compared [Synapomorphy Numbering as in Figure 6 and Figure 7]	Target Model	Compared Models	RMSD Value	
			RNAssess Web Server (Number of Atoms Compared)	PyMol (Number of Atoms Compared)
L(A) [s6]	*Fromundus pygmaeus*	*Thyreocoris scarabaeoides* *Stibaropus indonesicus* *Macroscytus badius* *Adrisa romani* *Pseudoscoparipes fraterculus* *Cydnus aterrimus* *Chilocoris piceus*	0.20 (453) 0.10 (453) 0.19 (453) 2.11 (288) 2.14 (288) 2.11 (288) 0.88 (194)	0.196 (453) 0.098 (453) 0.186 (453) 0.173 (453) 0.186 (453) 0.156 (453) 0.906 (424)
L(C) [s7]	*Thyreocoris scarabaeoides*	*Adrisa romani* *Pseudoscoparipes fraterculus*	0.66 (455) 0.20 (455)	0.660 (455) 0.198 (455)
L(D) [s8]	*Fromundus pygmaeus*	*Cyrtomenus emarginatus* *Macroscytus badius*	0.76 (389) 0.79 (389)	0.760 (390) 0.785 (390)
L(E) [s9]	*Fromundus pygmaeus*	*Stibaropus indonesicus* *Macroscytus badius* *Cyrtomenus emarginatus* *Adrisa romani* *Pseudoscoparipes fraterculus* *Ochetostethomorpha secunda*	0.73 (483) 0.32 (483) 0.25 (483) 0.33 (483) 0.37 (449) 0.70 (416)	0.742 (511) 0.388 (511) 0.330 (511) 0.401 (511) 0.445 (506) 0.746 (502)
L(E) [s10]	*Adomerus biguttatus*	*Parastrachia japonensis* *Thyreocoris scarabaeoides* *Amaurocoris curtus*	0.83 (416) 0.72 (449) 0.21 (449)	0.848 (445) 0.718 (449) 0.209 (449)

**Table 7:** RMSD values for the specific tertiary structure of the LVR L subregions, which can serve as potential morpho-molecular autapomorphies for a target model against which all other taxa were compared. The higher value from the calculation containing more atoms was deemed conclusive (marked * for results in RNAssess web server, and ** for results in PyMol).

Subregion Compared [Autapomorphy Numbering as in Figure 6 and Figure 7]	Target Model against which All Other Species Were Compared	Range of RMSD Values
L(A) [a5] L(A) [a6] L(B) [a4] L(B) [a7] L(C) [a8] L(E) [a1]	*Amaurocoris curtus*	1.1 **–11.5 **
	*Amnestus zacki*	10.3 **–11.7 **
	*Thyreocoris scarabaeoides*	3.7 **–10.8 *
	*Amnestus zacki*	3.9 **–18.4 **
	*Amnestus zacki*	4.4 **–6.9 **
	*Thaumastella elizabethae*	2.7 *–11.3 **
L(E) [a3]	*Cydnus aterrimus*	1.1 **–11.9 **
L(E) [a9]	*Amnestus zacki*	6.7 **–11.9 **
L(E) [a10]	*Chilocoris piceus*	3.9 **–10.7 **
L2 [a2]	*Thaumastella elizabethae*	1.1 **–4.8 **
L2 [a11]	*Chilocoris piceus*	2.8 **–5.7 **

**Figure 6:**
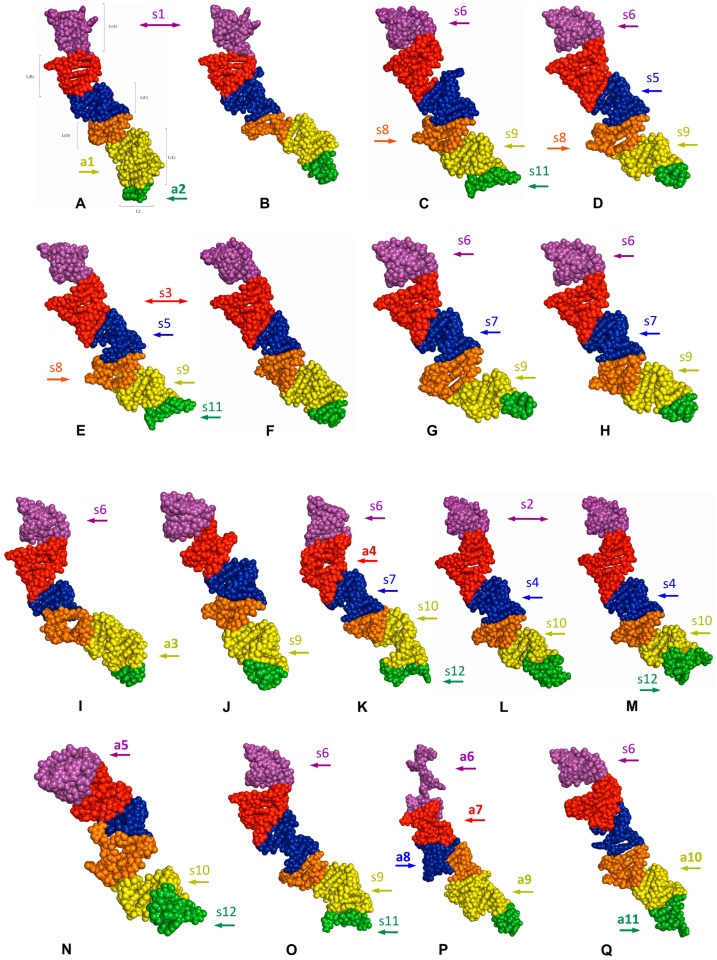
The predicted tertiary structure models of the LVR L. (**A**) *Thaumastella elizabethae* (Thauma-stellidae, outgroup). (**B**) *Garsauria aradoides* (Cydnidae: Garsauriinae). (**C**) *Fromundus pygmaeus* (Cydnidae: Cydninae). (**D**) *Macroscytus badius* (Cydnidae: Cydninae). (**E**) *Cyrtomenus emarginatus* (Cydnidae: Cydninae). (**F**) *Rhytidoporus indentatus* (Cydnidae: Cydninae). (**G**) *Adrisa romani* (Cydnidae: Cydninae). (**H**) *Pseudoscoparipes fraterculus* (Cydnidae: Cydninae). (**I**) *Cydnus aterrimus* (Cydnidae: Cydninae). (**J**) *Ochetostethomorpha secunda* (Cydnidae: Sehirinae). (**K**) *Thyreocoris scarabaeoides* (Thyreocoridae). (**L**) *Parastrachia japonensis* (Parastrachiidae). (**M**) *Adomerus biguttatus* (Cydnidae: Sehirinae). (**N**) *Amaurocoris curtus* (Cydnidae: Amaurocorinae). (**O**) *Stibaropus indonesicus* (Cydnidae: Cephalocteinae). (**P**) *Amnestus zacki* (Cydnidae: Amnestinae). (**Q**) *Chilocoris piceus* (Cydnidae: Cydninae). The arrows corresponding in colour to the particular LVR L subregion indicate the fragments that can serve as potential morpho-molecularly derived characters: (s1–s14) synapomorphies, (a1–a11) autapomorphies [for symbols explanation see Table 5 and Table 6]. Sequences are aligned with the outgroup (*T. elizabethae*) sequence.

**Figure 7:**
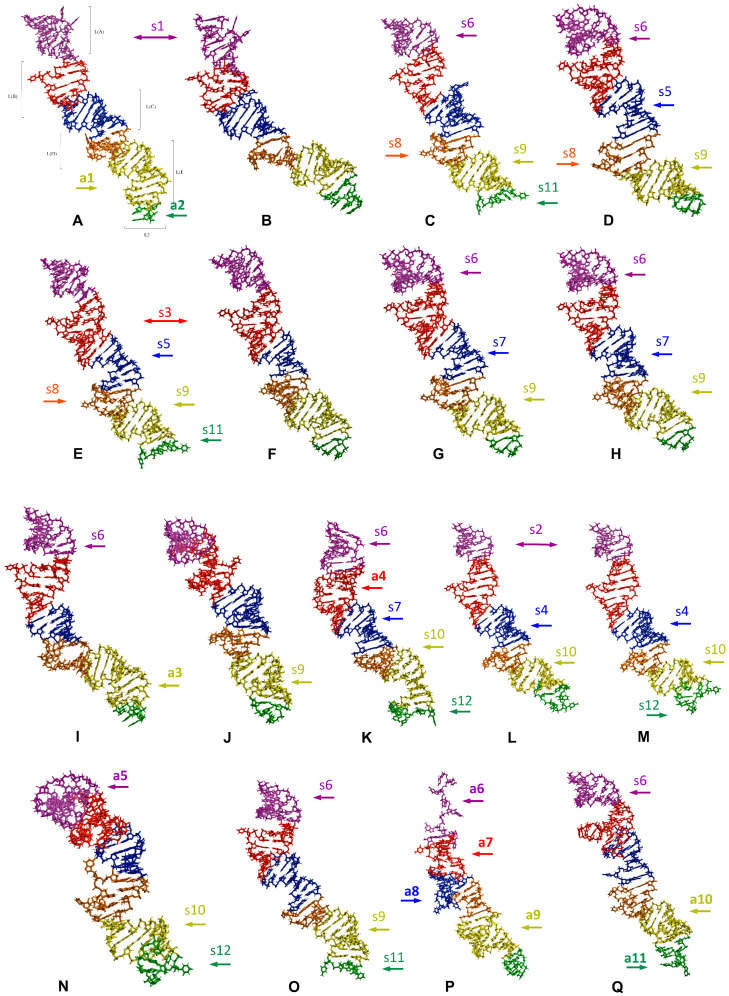
The predicted tertiary structure models of the LVR L, showing ring structures and hydrogen bonds. (**A**) *Thaumastella elizabethae* (Thaumastellidae, outgroup). (**B**) *Garsauria aradoides* (Cydnidae: Garsauriinae). (**C**) *Fromundus pygmaeus* (Cydnidae: Cydninae). (**D)** *Macroscytus badius* (Cydnidae: Cydninae). (**E**) *Cyrtomenus emarginatus* (Cydnidae: Cydninae). (**F**) *Rhytidoporus indentatus* (Cydnidae: Cydninae). (**G**) *Adrisa romani* (Cydnidae: Cydninae). (**H**) *Pseudoscoparipes fraterculus* (Cydnidae: Cydninae). (**I**) *Cydnus aterrimus* (Cydnidae: Cydninae). (**J**) *Ochetostethomorpha secunda* (Cydnidae: Sehirinae). (**K**) *Thyreocoris scarabaeoides* (Thyreocoridae). (**L**) *Parastrachia japonensis* (Parastrachiidae). (**M**) *Adomerus biguttatus* (Cydnidae: Sehirinae). (**N**) *Amaurocoris curtus* (Cydnidae: Amaurocorinae). (**O**) *Stibaropus indonesicus* (Cydnidae: Cephalocteinae). (**P**) *Amnestus zacki* (Cydnidae: Amnestinae). (**Q**) *Chilocoris piceus* (Cydnidae: Cydninae). The arrows corresponding in colour to the particular LVR L subregion indicate the fragments that can serve as potential morpho-molecularly derived characters: (s1–s14) synapomorphies, (a1–a11) autapomorphies (for an explanation of the symbols, see Table 5 and Table 6). Sequences are aligned with the outgroup (*T. elizabethae*) sequence.

The predicted tertiary structures of the LVR L subregions for all 17 analysed species are presented in Figure 6 and Figure 7. All subregions that could serve as potential morpho-molecular autapomorphies or synapomorphies are indicated by coloured arrows corresponding to particular LVR L subregions (Figure 6 and Figure 7). All other subregions are plesiomorphic (as defined by Lis [1]) in terms of their number of nucleotides and their secondary and tertiary structure.

## 3. Discussion

### 3.1. Potential Plesiomorphies and Apomorphies in LVR L Secondary Structures

The present analysis of LVR L secondary structures confirmed the occurrence of predefined plesiomorphic conditions for nucleotide numbers in specific subregions within Pentatomoidea [1]. These plesiomorphies were found in all examined subregions except for the LE subregion, in which they were absent in species from the family Cydnidae and its closest relatives (Parastrachiidae, Thaumastellidae, and Thyreocoridae). Therefore, we identified the LVR LE subregion as the most diverse subregion within the superfamily Pentatomoidea, which was tentatively suggested by previous studies [1].

As previous studies focused on the superfamily Pentatomoidea [1], our analyses validated several synapomorphies and autapomorphies in nucleotide numbers within specific subregions.

### 3.2. Potential Synapomorphies and Autapomorphies in LVR L Tertiary Structure

The RMSD values computed for the species with identical secondary structures for each of the LVR L subregions indicated that certain structures could be classified as morpho-molecular derived apomorphies (autapo- or synapomorphies), while others could not.

The LA subregion exhibited considerable variation in terms of nucleotide numbers (11–17 nucleotides in total) and had six distinct nucleotide schemes. The results derived from the secondary and tertiary structure predictions indicate the presence of three possible morpho-molecular synapomorphies within this subregion. The first synapomorphy pertains to structures in *T. elizabethae* (Thaumastellidae) and *G. aradoides* (Cydnidae: Garsauriinae). The second was observed in *A*. *biguttatus* (Cydnidae: Sehirinae) and *P. japonensis* (Parastrachiidae). The third synapomorphy was observed in the secondary and tertiary structures. It was identified in eight species, one from Thyreocoridae and the remaining seven from Cydnidae. These Cydnidae species belong to two different subfamilies: Cephalocteinae (*S. indonesicus*) and Cydninae (six species). The RMSD values derived by analysing the tertiary structure of the LVR LA subregion indicated two distinct morpho-molecular autapomorphies: one for the subfamily Amnestinae (*A. zacki*) and the other for Amaurocorinae (*A. curtus*).

Like the LA subregion, the LB subregion had a notable variation in nucleotide numbers (11–19 nucleotides) with 6 nucleotide arrangements. Despite having the largest number of secondary structure types compared to other subregions (up to 11), only one morpho-molecular synapomorphy was identified. This was identified in two species representing the tribe Geotomini (of the subfamily Cydninae): *C. emarginatus* and *R. indentatus*. The RMSD values calculated for the tertiary structures of the LB subregion indicated the presence of two morpho-molecular autapomorphies. One was found in *A. zacki* (a consensus species of the subfamily Amnestinae) and the other in *T. scarabaeoides* (a consensus species of the family Thyreocoridae).

In the LC subregion, six nucleotide arrangements and ten types of secondary structures were identified. However, this subregion only demonstrated three morpho-molecular synapomorphies in the tertiary structures and one morpho-molecular autapomorphy. Synapomorphies within the tertiary structures of this subregion were observed in *A. biguttatus* (Cydnidae: Sehirinae) and *P. japonensis* (Parastrachiidae); *M. badius* and *C. emarginatus* (both representing the tribe Geotomini in the subfamily Cydninae of Cydnidae); *T. scarabaeoides* (Thyreocoridae), *A. romani* and *P. fraterculus* (from the tribe Geotomini). Only one morpho-molecular autapomorphy was identified in *A. zacki*, a consensus species for the subfamily Amnestinae of the Cydnidae.

The LD subregion exhibited significant diversity in nucleotide numbers, with six possible arrangements. Six secondary structures were also identified. However, only one morpho-molecular synapomorphy was present in the tertiary structures. This synapomorphy was seen in three species of the tribe Geotomini: *F. pygmaeus*, *C. emarginatus*, and *M. badius*. No morpho-molecular autapomorphies were detected within this subregion.

In the LE region, seven nucleotide arrangements and seven secondary structure types were identified. The results derived from the secondary and tertiary structure predictions indicated the presence of two morpho-molecular synapomorphies and four autapomorphies. The first morpho-molecular synapomorphy was detected for a group of seven Cydnidae species from three different subfamilies: Sehirinae (*O. secunda*), Cephalocteinae (*S. indonesicus*), and Cydninae (five species representing the tribe Geotomini *sensu lato*). The second synapomorphy was identified in four species. However, only two belonged to the family Cydnidae: *A. biguttatus* from the subfamily Sehirinae and *A. curtus* from the subfamily Amaurocorinae. The remaining two species were not members of the family Cydnidae and were from two closely related families: *P. japonensis* (a consensus species for the Parastrachiidae) and *T. scarabaeoides* (a consensus species for the Thy-reocoridae).

The LE subregion contained the highest number of autapomorphies within the LVR L, with four detected. These were identified in *T. elizabethae* (Thaumastellidae), *A. zacki* (Cydnidae: Amnestinae), and *C. piceus* and *C. aterrimus* (both representing the tribe Cydnini of the subfamily Cydninae).

The L2 subregion was the most constant. Lis [1] identified four nucleotides that were plesiomorphic and six that were symplesiomorphic. In the current study, *T. elizabethae* (Thaumastellidae) and *C. piceus* (Cydnidae: Cydninae: Cydnini) had three and seven nucleotides as autapomorphies, respectively.

### 3.3. Systematic Position of the Family Thaumastellidae

The current analyses of the LVR L of 18S rRNA secondary and tertiary structures support earlier findings [1,4,9,10,33], indicating that Thaumastellidae is not a member of the family Cydnidae, irrespective of its internal classification, and should be recognised as a distinct Pentatomoidea family.

The distinctiveness of this family in the ‘cydnoid’ complex, as specified by Lis et al. [4], was verified by the results of the RSMD calculations for the tertiary structures of all the examined species. *T. elizabethae* (a consensus species of Thaumastellidae) exhibited distinctive morpho-molecular autapomorphies in two subregions: LE and L2.

Identifying a synapomorphy between *T. elizabethae* and *G. aradoides* was a significant discovery during the structure analyses. This aspect has not been previously studied in the subfamily Garsauriinae of Cydnidae, nor have these species’ genetic sequences been analysed. Due to significant differences in the RMSD values obtained from RNAsses and PyMol calculations, future analyses should concentrate on the molecular relationships between Thaumastellidae representatives and species within the subfamily Garsauriinae.

### 3.4. Classification of the Family Cydnidae versus Morpho-Molecular Apomorphies in the LVR L

The RMSD values calculated for the Cydnidae species with equivalent secondary structures in each of the LVR L subregions show that certain subregions can be identified as morpho-molecular apomorphies in the tertiary structures.

The analyses revealed no synapomorphies in the family Cydnidae, including all currently recognised subfamilies. This lack of synapomorphies was observed in the primary, secondary, and tertiary structures of the studied 18S rRNA region. In addition, no autapomorphy was identified within the region that could distinguish Cydnidae as a well-defined monophyletic group within the ‘cydnoid’ complex. These results support previous hypotheses regarding the non-monophyletic origin of this family [4,11,12,20].

The analysis of the morpho-molecular synapomorphies for the subfamily Sehirinae as a monophyletic group revealed two significant discrepancies that challenge the current classification of this subfamily and the family Cydnidae. Firstly, the data indicate a distinction between a group of two species, *Ochetostethus opacus* and *Ochetostethomorpha secunda*, from other species of the subfamily Sehirinae. This is because they have four nucleotides in the L2 subregion, equal to that of species representing the two other subfamilies: Cydninae and Cephalocteinae. In contrast, the remaining Sehirinae species have six nucleotides in this subregion. The uniqueness of this group within the subfamily Sehirinae is further indicated by the presence of a synapomorphy in subregion LE in *O. secunda* (the consensus species for the ochetostethan facies of spermatheca) and several non-sehirine species representing the subfamilies Cydninae and Cephalocteinae.

Consistent with previous molecular studies on the superfamily Pentatomoidea [1,4], our analyses revealed synapomorphies in the LA and LC subregions of Sehirinae and Parastrachidae. However, the synapomorphy identified in the tertiary structure of the LE subregion observed in *A. biguttatus* (Cydnidae: Sehirinae), *P. japonensis* (Parastrachiidae), *T. scarabaeoides* (Thyreocoridae), and *A. curtus* (Cydnidae: Amaurocorinae), was not supported by the results of the phylogenetic analysis.

As in the case of the subfamily Sehirinae, we could not identify morpho-molecular synapomorphies for the entire subfamily Cydninae in any of the analysed subregions. Of the nine analysed Cydninae species, morpho-molecular synapomorphies were only identified in the LA subregions of six species, the LD subregions of three species, and the LE subregions of five species. In addition to the members of Cydninae, several species from other subfamilies or related families are also included in the groups defined by a particular synapomorphy. The LA subregion synapomorphy includes two additional species: one from the subfamily Cephalocteinae (*S. indonesicus*) and *T. scarabaeoides* of the family Thyreocoridae. For the LE subregion, in addition to five species from Cydninae, one species of the subfamily Cephalocteinae (*S. indonesicus*) and one species of the subfamily Sehirinae (*O. secunda*) are linked by this synapomorphy.

The positioning of the Cephalocteinae species (*S. indonesicus*) on the phylogenetic tree indicates a distinct correlation with the species of the tribe Geotomini *sensu lato* (subfamily Cydninae). These findings supplement the current results. This is further supported by the number of nucleotides present in the subregions of the LVR L of *S. indonesicus*, which matches with *F. pygmaeus* (Geotomini) across all six subregions and shares similarities with other Geotomini tribe species in five sub-regions. Moreover, it has the same type and facies of spermatheca as representatives of this tribe. The Cephalocteinae subfamily does not demonstrate any autapomorphy, unlike the subfamilies of Amaurocorinae and Amnestinae. Therefore, it might be suitable to categorise the subfamily species as part of the tribe Geotomini or a separate tribe within the subfamily Cydninae, as previously proposed by Wagner [37].

The findings related to the subfamily Cydninae indicate that its two tribes, Geoto-mini *sensu lato* and Cydnini, are heterogeneous. This is particularly evident in the tribe Cydnini, which comprises two separate, distinguishable groups. The first includes all *Chilocoris* species and *Parachilocoris minutus*, while the second consists of the tribe’s type genus and species, *Cydnus aterrimus*.

The consensus species (*C. piceus*) of the first group is distinct from *C. aterrimus* in several molecular characteristics. The length of the LVR L region in *C. piceus* (81 nucleotides) differs from *C. aterrimus* (73 nucleotides) and is consistent with the length seen in other species of the Cydninae, Sehirinae, Amaurocorinae, Amnestinae, and Parastrachiidae subfamilies. Additionally, both groups exhibited different nucleotide numbers in the LC, LD, LE, and L2 subregions, with the discrepancy in L2 being particularly noteworthy. *C. piceus* had seven nucleotides in this subregion, a characteristic unique to it (autapomorphy), while *C. aterrimus* only exhibited four nucleotides, a feature common to the entire Pentatomoidea (a plesiomorphic state, [1]). The LVR L subregions, specifically LB, LC, LD, LE, and L2 in species from both Cydnini groups, exhibited distinct secondary structures. A shared characteristic (synapomorphy) for both groups was only detected in the secondary and tertiary structures of the LA subregion.

Furthermore, different morpho-molecular autapomorphies were detected for each group. Two autapomorphies were identified in *C. piceus* tertiary structures in the LE and L2 subregions, while one was identified in *C. aterrimus* in the LE subregion. The differences between *C. aterrimus* and *C. piceus* were significant due to differences in nucleotide numbers and secondary structures. The recovered phylogenetic relationship between these two groups suggests that they are distantly related. *C. aterrimus* unexpectedly belongs to the tribe Geotomini *sensu lato*, while *C. piceus* (a consensus species of the remaining Cydnini) is the closest relative to the subfamily Amnestinae. An inferred potential close relationship between species of the subfamily Amnestinae and species of the *Chilocoris* and *Parachilocoris* genera (Cydninae: Cydnini) was previously suggested following an analysis of wing stridulitrum across the family Cydnidae [38]. Together, these findings suggest that the two groups that comprise Cydnini (*Cydnus* versus *Chilocoris* + *Parachilocoris*) are not phylogenetically related and probably should not be grouped in the same tribe.

The subfamily Amaurocorinae shares certain characteristics with species of the subfamilies Cydninae and Sehirinae, particularly regarding the number of nucleotides within various LVR L subregions. These similarities are also evident in the secondary structures of multiple subregions. Comparable secondary structure patterns were also found in species belonging to two other families: Thyreocoridae and Parastrachiidae. The presence of morpho-molecular synapomorphies in tertiary structures suggests a possible relationship between this subfamily and species of the subfamily Sehirinae, as well as two other families related to the Cydnidae: Thyreocoridae and Parastrachiidae. However, the status of Amaurocorinae as a subfamily could be justified by the existence of its morpho-molecular autapomorphy in the LA subregion.

The subfamily Amnestinae was most distinct within the Cydnidae. It exhibited the greatest number of unique morpho-molecular autapomorphies, identified in four out of the six LVR L subregions: LA, LB, LC, and LD. In addition, the species in this subfamily did not exhibit any morpho-molecular synapomorphies with other subfamilies within the Cydnidae or with closely related families, such as Thyreocoridae and Parastrachiidae.

One morpho-molecular characteristic was identified in the subfamily Garsauriinae, a synapomorphy in the LA subregion shared with *T. elizabethae* (Thaumastellidae). This subfamily shared no synapomorphies with any of the analysed taxa of the Cydnidae, Thyreocoridae, or Parastrachiidae families. Therefore, the relationship between this subfamily and others within the family Cydnidae remains unclear, as has been previously suggested [19].

## 4. Materials and Methods

### 4.1. Selection of Taxa

This study analysed the V4 hypervariable region sequences of 18S rDNA across 45 species, representing all four families of the ‘cydnoid’ complex within the superfamily Pentatomoidea [4]. Fifteen sequences were obtained directly from GenBank (Table S3), and 30 were newly sequenced (Table S4). The widely accepted concept of the family Cydnidae [1,9,10,19,21,25,27,29,30,31,32,33] was considered the baseline for all analyses. The analysed taxa included representatives of the Thaumastellidae (two species), Parastrachiidae (two species), Thyreocoridae (two species), and Cydnidae (39 species). The latter included members of all six currently recognised subfamilies [19,21]: Amaurocorinae (one species), Amnestinae (three species), Cephalocteinae (one species), Cydninae (twenty-five species), Garsauriinae (one species), and Sehirinae (seven species) (Table S1).

This is the first time the family Cydnidae has been represented by species belonging to all subfamilies and almost all their tribes. Only one tribe, the Cephalocteinae, was absent from the analysis. Previously, only taxa of two subfamilies, Sehirinae and Cydninae, have been used in molecular studies [1,4,8,9,10,11,12,15,16,36,39,40,41,42,43,44,45,46,47,48].

To verify the sequence variability of the species representing the two tribes recognised as non-monophyletic by Pluot-Sigwalt and Lis [19], namely the Geotomini (subfamily Cydninae) and Sehirini (subfamily Sehirinae), the species were assigned to the groups of spermathecal types and facies recovered within these two tribes (Table 1 and Table S1). To identify the monophyletic groups and their ‘consensus species’, which are essential for predicting secondary and tertiary structures (see Section 4.5), representatives of the Thaumastellidae family were considered outgroup species [1] during the relationship analysis (Figure A1).

The taxa names, specimens’ geographic origins, collectors’ names (if available), the University of Opole (Poland) sample numbers (if relevant), and the accession numbers for sequences we deposited into GenBank and those obtained directly from GenBank are provided in Tables S3 and S4.

### 4.2. DNA Extraction

Genomic DNA was extracted from the thorax muscle tissues of ethanol-preserved specimens using the DNeasy Tissue Kit (QIAGEN Inc., Santa Clara, CA, USA) per the manufacturer’s guidelines. Sample residues were placed in tubes containing 96% ethanol and cryopreserved in a freezer at the Institute of Biology, University of Opole (University of Opole sample numbers are listed in Table S3).

### 4.3. PCR Amplification, Purification and Sequencing

To amplify the 18S rDNA fragments containing the V4 hypervariable regions via PCR, a 25 μL reaction volume composed of 12.5 μL reaction buffer (PCR Mix Plus, A&A Biotechnology, Gdańsk, Poland), 1 μL of DNA template, 0.5 μL of each primer, and 10.5 μL ultrapure water was used. A set of primers (5′⟶3′) was used for the amplification [49,50]: 3F (forward: GTT CGA TTC CGG AGA GGG A)–18Sbi (reverse: GAG TCT CGT TCG TTA TCG GA).

The PCR reactions were performed according to the protocol described by Lis et al. [2]. The amplification consisted of 36 cycles of denaturation at 93 °C for 1 min, annealing at 59 °C for 1 min, and extension at 72 °C for 40 s. The amplification was initialised via incubation at 93 °C for 2 min and a final extension at 72 °C for 5 min. The quality of the final PCR products was assessed using 1% agarose gel electrophoresis. Successful samples were purified using the Clean-Up purification kit (A&A Biotechnology, Gdańsk, Poland).

All experimental PCR runs were performed simultaneously with negative controls (i.e., reactions without template DNA). Sequencing was performed at GENOMED S.A. (Warszawa, Poland) and A&A Biotechnology (Gdańsk, Poland). The sequences obtained were verified by BLAST searches to confirm that the results were not due to contaminants. All newly obtained DNA sequences were deposited in GenBank (OR691631–OR691660). Their accession numbers are given in Tables S3 and S4.

### 4.4. Reconstruction of 18S rRNA Secondary Structure Models

The 18S rRNA secondary structure of two species in the family Cydnidae, *Fromundus pygmaeus* (Dallas, 1851) and *Adomerus biguttatus* (Linnaeus, 1758), was designed according to the universal model of the gene provided for insects [3]. Some necessary modifications and reinterpretations already proposed for Heteroptera have been described in detail by Lis [1]. These species represented the two largest subfamilies of the Cydnidae (Cydninae and Sehirinae, respectively), and both had their 18S rDNA sequenced [1,16] and deposited in GenBank (*F. pygmaeus*, GenBank accession no.: KJ535871; *A. biguttatus*, GenBank accession no.: KY886253).

The hypervariable region numbering and numbering scheme used for the LVRs was adopted from the works of Lis [1], Yu et al. [15], Wu et al. [16], and Neefs et al. [51].

### 4.5. Prediction of LVR L Secondary Structure

Secondary LVR L structures of all analysed species were predicted using RNAstructure ver. 6.3 [52]. A three-step procedure was used for comparative sequence analysis based on the study by Lis [1]. First, structure predictions were made for each species separately. Then, if only two species were identified as strictly monophyletic, a secondary structure common to both sequences was predicted. Secondary structures common to three or more sequences were calculated if the identified monophyla were represented by three or more species [52]. The species selected by the RNAstructure ver. 6.3 [52] were those that had secondary structures common to two or more sequences; these species were considered ‘consensus species’ for these sequences (Table 1 and Table S1).

The results of the phylogenetic analysis of the 45 species (Figure A1) were used to determine the number of steps to be followed to predict the secondary structures, but not to suggest any changes in the classification of the family Cydnidae. To mitigate the influence of missing data from incomplete sequences, the sequences were aligned using ClustalW with default parameters in the MEGAX software v. 10.2.6 [53] and then truncated at both ends. A Maximum Likelihood tree was generated via IQ-TREE [54] on the web server [33] using 10,000 replicates and the ultrafast bootstrap method [55]. The resulting tree was visualised and edited using the online tool iTOL v5 [56]. It was prepared for publication using CorelDRAW 21.

The subdivision of the secondary structures into subregions was performed according to the method used by Lis [1,2]. All secondary structures were visualised using the Structure Editor in RNAstructure ver. 6.3 [52].

### 4.6. Prediction of 18S rRNA Tertiary Structures

The 18S rRNA tertiary structure was predicted using the 3dRNA v2.0 Web Server (http://biophy.hust.edu.cn/new/3dRNA, accessed on 15 January 2022) [57]. As with the secondary structures, the procedures described in Lis [1] and Lis et al. [2] were followed. The tertiary structure of 18S rRNA was predicted for two species: *Fromundus pygmaeus* (Cydnidae: Cydninae) and *Adomerus biguttatus* (Cydnidae: Sehirinae). The predictions used the 18S rDNA sequences already deposited in GenBank *(F. pygmaeus*, GenBank accession no.: KJ535871; *A. biguttatus*, GenBank accession no.: KY886253). The structural images were visualised using PyMol software ver. 2.4.0 [35].

### 4.7. Prediction of LVR L Tertiary Structure

RNAComposer (http://rnacomposer.ibch.poznan.pl, accessed on 18 August 2023), a fully automated RNA structure modelling server, was used to predict the tertiary structure of LVR L [58,59]. The RNAComposer method allows RNA structures up to 500 nucleotides long to be constructed with high accuracy and accounts for the possible occurrence of pseudoknots [58,59]. Twenty 3D models were generated for each LVR L sequence. The best model with the lowest free energy was selected for further investigation. All tertiary structure images were visualised using PyMol software ver. 2.4.0 [35].

### 4.8. Concept of the Morpho-Molecular Structures Potentially Serving as Derived Characters

The concept of morpho-molecular apomorphies (autapomorphies and synapomorphies) of nucleotide sequences in the predicted secondary structures was adapted from Ouvrard et al. [14], Yu et al. [15], and Xie et al. [60]. The definition of plesiomorphic states within Pentatomoidea in terms of the number of nucleotides in each LVR L region and the secondary and tertiary structures was adapted from Lis [1].

The strategy for identifying morpho-molecular derived characters in the predicted tertiary structures was based on the methods proposed by Lis [1] and Lis et al. [2]. Based on this [1], all morpho-molecular tertiary structures were identified based on their degree of uniqueness [61]. Only the tertiary structures whose uniqueness was confirmed at the secondary structure level were considered derived characters (apomorphies) and were used for further analysis. Furthermore, only structures that had a subregion-specific nucleotide arrangement (Figure S2) and different secondary (File S2) and tertiary (Figure 6 and Figure 7) structures were considered potential morpho-molecular autapomorphies.

The uniqueness of the tertiary structures was confirmed using Root-Mean-Square Deviation (RMSD) values, a widely accepted method for comparing 3D structures [34,35,61,62]. The RMSD computation aligns atoms in the predicted and reference structures, indicating tertiary structure likeness [34,35,61,62]. RMSD is equal to 0 for theoretically identical structures. As the dissimilarity between two structures increases, so does the RMSD value [34,35,61,62]. RMSD values were calculated for the LVR L tertiary structures of all species using PyMol [35] and the RNAssess web server [34]. Additionally, the Interaction Network Fidelity (INF), Deformation Profile (DP), and *p*-value coefficients were calculated to determine the similarity between structures with comparable RMSD values more accurately [34].

For the specific tertiary structures of the LVR L subregions, any RMSD value less than 1Å between the compared model sequences was considered sufficient to identify them as potential morpho-molecular synapomorphies for the two or more species analysed [63,64]. If there was a difference between the RMSD value calculated in PyMol [35] and that computed by the RNAssess web server [34], the value from the calculation containing more atoms was deemed conclusive. Potential morpho-molecular autapomorphies in a particular subregion were deemed reliable only if a calculated RMSD greater than 1Å existed between all analysed structures and the compared model.

## 5. Conclusions

1. Comparisons of the predicted tertiary structures of the LVR L of the 18S rRNA in species representing all presently recognised and accepted subfamilies and tribes within the family Cydnidae revealed inconsistencies in their classifications.
2. The present comparative analyses of the LVR L of the 18S rRNA secondary and tertiary structures support earlier findings that irrespective of its internal classification, Thaumastellidae is not a member of the family Cydnidae and should be recognised as a distinct Pentatomoidea family.
3. The analysis did not identify one synapomorphy that was present across all presently acknowledged subfamilies of Cydnidae. This absence was observable in the primary, secondary, and tertiary structures of the studied 18S ribosomal RNA region. Furthermore, no autapomorphy was detected in the examined region to differentiate Cydnidae as a monophyletic group within the ‘cydnoid’ complex. These findings are consistent with previous hypotheses suggesting that the origin of this family is non-monophyletic.
4. The predicted secondary and tertiary structures of the LVR L of the 18S rRNA of the family Parastrachiidae and the subfamily Sehirinae (Cydnidae) confirm their close relationship, highlighted by the several morpho-molecular synapomorphies shared between their LVR L subregions.
5. Two notable groups of species in the subfamily Sehirinae were found to be unrelated. These groups challenged the classification currently in use for this subfamily. One group displayed ochetostethan facies of spermatheca, which significantly differed in regard to their morpho-molecular data from species representing the sehiran facies of spermatheca within the subfamily. These findings indicate that the subfamily may need to be divided into at least two tribes. However, further supportive analyses incorporating alternative mitochondrial and nuclear genes must be conducted to address this further.
6. The subfamily Cephalocteinae displayed a clear correlation with the species of the tribe Geotomini across several morpho-molecular data. It does not possess any distinctive morpho-molecular autapomorphy and exhibits the same type and facies of spermatheca as representatives of the tribe. Therefore, the subfamily may be classified as part of the tribe Geotomini or as a distinct tribe within the subfamily Cydninae.
7. The relationship between two groups from the tribe Cydnini (*C. aterrimus* and *C. piceus*) suggests they are distantly related. *C. aterrimus* was found to be closely related to the tribe Geotomini, while *C. piceus* (the consensus species of the remaining Cydnini) appeared to be the closest relative to the subfamily Amnestinae. These findings imply that these two groups are not phylogenetically related. Therefore, it is likely inappropriate to categorise them as belonging to the same tribe.
8. The subfamily status of Amaurocorinae was confirmed based on the morpho-molecular autapomorphy in the LA subregion of the LVR L, despite its relation to species of the subfamilies Cydninae and Sehirinae, as well as those of the families Thyreocoridae and Parastrachiidae in terms of morpho-molecular LVR L characters.
9. The Amnestinae is the most distinct subfamily in the Cydnidae family due to its numerous morpho-molecular autapomorphies. Additionally, the group’s species do not show morpho-molecular synapomorphies with other subfamilies within the Cydnidae or closely related families, such as Thyreocoridae and Parastrachiidae.
10. The subfamily Garsauriinae shared only one morpho-molecular synapomorphy with the other studied taxa, specifically with the family Thaumastellidae. Additionally, no synapomorphies were found in this subfamily with any other taxa of Cydnidae, Thyreocoridae, or Parastrachiidae. Therefore, the relationship of this subfamily to others within the family Cydnidae remains unclear.

## Figures and Tables

**Table 1:** The number of nucleotides of the hypervariable region V4, the length-variable region L (LVR L), and the L2 subregion of the LVR L in the *18S rRNA* of the ‘consensus species’ for specific groups of taxa. The ‘consensus species’ for family, subfamily or facies are marked with *; the ‘consensus species’ for the clades within the specific tribe are marked with **. Each analysed species of the Cydnidae were assigned to the particular group of spermathecal types and facies recovered within the family [19].

Family	Subfamily	Spermathecal Types/Facies (According to [19])	Species	Number of Nucleotides		
				V4	LVR L	L2
Cydnidae	Amaurocorinae	Amaurocorinae type	*Amaurocoris curtus* (Brullé, 1838) *	316	73	6
	Amnestinae	Amnestinae type	*Amnestus zacki* Mayorga & Cervantes, 2009 *	324	73	6
	Garsauriinae	Garsauriinae type	*Garsauria aradoides* Walker, 1868 *	317	74	6
	Cephalocteinae	Cydnoid type Geotoman facies	*Stibaropus indonesicus* J.A. Lis, 1991 *	317	74	4
	Cydninae	Cydnoid type Geotoman facies	*Fromundus pygmaeus* (Dallas, 1851) **	317	74	4
			*Macroscytus badius* (Walker, 1867) **	317	74	4
			*Cyrtomenus emarginatus* Stål, 1862 **	317	74	4
			*Rhytidoporus indentatus* Uhler, 1877 **	317	74	4
		Cydnoid type Adrisan facies	*Adrisa romani* J.A. Lis, 1994 *	316	73	4
		Cydnoid type Scoparipan facies	*Pseudoscoparipes fraterculus* J.A. Lis, 1994 *	316	73	4
		Cydnoid type Cydnan facies	*Chilocoris piceus* Signoret, 1884 **	325	81	7
			*Cydnus aterrimus* (Forster, 1771) **	316	73	4
	Sehirinae	Cydnoid type Sehiran facies	*Adomerus biguttatus* (Linnaeus, 1758) *	316	73	6
		Cydnoid type Ochetostethan facies	*Ochetostethomorpha secunda* J.A. Lis & B. Lis, 2014 *	316	73	4
Parastrachiidae	–	–	*Parastrachia japonensis* (Scott, 1880) *	316	73	6
Thyreocoridae	Thyreocorinae	–	*Thyreocoris scarabaeoides* (Linnaeus, 1758) *	316	74	6
Thaumastellidae (outgroup)	–	–	*Thaumastella elizabethae* Jacobs, 1989 *	318	75	3

**Table 2:** The number of nucleotides of the hypervariable region V4, the length-variable region L (LVR L), and the L2 subregion of the LVR L in the *18S rRNA* of the analysed higher taxa.

Region or Subregion	Number of Nucleotides	Family	Subfamily and Tribe
V4	315	Thaumastellidae	–
	316	Cydnidae	Amaurocorinae
			Cydninae: Geotomini *s. lato* [part]
			Sehirinae: Sehirini *s. lato* [part]
		Parastrachiidae	–
		Thyreocoridae	Corimelaeninae
			Thyreocorinae
	317	Cydnidae	Garsauriinae
			Cephalocteinae
			Cydninae: Geotomini *s. lato* [part]
	318	Thaumastellidae	–
	324	Cydnidae	Amnestinae
	325	Cydnidae	Cydninae: Cydnini [part]
LVR L	72	Thaumastellidae	–
	73	Cydnidae	Amaurocorinae
			Amnestinae
			Cydninae: Geotomini *s. lato* [part]
			Cydninae: Cydnini [part]
			Sehirinae: Sehirini *s. lato*
		Parastrachiidae	–
	74	Cydnidae	Garsauriinae
			Cephalocteinae
			Cydninae: Geotomini *s. lato* [part]
		Thyreocoridae	Corimelaeninae
			Thyreocorinae
	75	Thaumastellidae	–
	81	Cydnidae	Cydninae: Cydnini [part]
L2	3	Thaumastellidae	–
	4	Cydnidae	Cephalocteinae
			Cydninae: Geotomini *s. lato*
			Cydninae: Cydnini [part]
			Sehirinae: Sehirini *s. lato* [part]
	6	Cydnidae	Amaurocorinae
			Garsauriinae
			Amnestinae
			Sehirinae: Sehirini *s. lato* [part]
		Parastrachiidae	–
		Thyreocoridae	Corimelaeninae
			Thyreocorinae
	7	Cydnidae	Cydninae: Cydnini [part]

**Table 3:** The nucleotide numbers of the subregions of the LVR L. The autapomorphies in nucleotide numbers for Thaumastellidae are indicated in red, Thyreocoridae in purple, *Chilocoris piceus* in green, *Cydnus aterrimus* in grey, and Amnestinae in blue. The plesiomorphic nucleotide number in each subregion is shown in yellow. The ‘consensus species’ for family, subfamily or facies are marked with *; the ‘consensus species’ for the clades within the specific tribe are marked with **.

Taxon Group	Species	Total Length	Number of Nucleotides of the LVR L Subregions					
			L2	LA (A1 + A2)	LB (B1 + B2)	LC (C1 + C2)	LD (D1 + D2)	LE (E1 + E2)
Thaumastellidae (outgroup)	*Thaumastella elizabethae* *	75	3	17 (9 + 8)	11 (6 + 5)	16 (8 + 8)	8 (5 + 3)	20 (10 + 10)
Cydnidae: Cydninae	*Cyrtomenus emarginatus* **	74	4	15 (8 + 7)	18 (10 + 8)	12 (6 + 6)	9 (6 + 3)	16 (8 + 8)
Cydnidae: Cydninae	*Rhytidoporus indentatus* **	74	4	15 (8 + 7)	18 (10 + 8)	10 (5 + 5)	11 (7 + 4)	16 (8 + 8)
Cydnidae: Cephalocteinae	*Stibaropus indonesicus* *	74	4	14 (7 + 7)	16 (9 + 7)	15 (8 + 7)	9 (6 + 3)	16 (8 + 8)
Cydnidae: Cydninae	*Fromundus pygmaeus* **	74	4	14 (7 + 7)	16 (9 + 7)	15 (8 + 7)	9 (6 + 3)	16 (8 + 8)
Cydnidae: Cydninae	*Macroscytus badius* **	74	4	14 (7 + 7)	19 (11 + 8)	12 (6 + 6)	9 (6 + 3)	16 (8 + 8)
Cydnidae: Cydninae	*Adrisa romani* *	73	4	14 (7 + 7)	16 (9 + 7)	14 (7 + 7)	9 (6 + 3)	16 (8 + 8)
Cydnidae: Cydninae	*Pseudoscoparipes fraterculus* *	73	4	14 (7 + 7)	16 (9 + 7)	14 (7 + 7)	9 (6 + 3)	16 (8 + 8)
Cydnidae: Cydninae	*Cydnus aterrimus* **	73	4	14 (7 + 7)	16 (9 + 7)	10 (5 + 5)	10 (7 + 3)	19 (9 + 10)
Cydnidae: Sehirinae	*Ochetostethomorpha secunda* *	73	4	12 (6 + 6)	18 (10 + 8)	14 (7 + 7)	9 (6 + 3)	16 (8 + 8)
Thyreocoridae	*Thyreocoris scarabaeoides* *	74	6	14 (7 + 7)	17 (10 + 7)	14 (7 + 7)	9 (6 + 3)	14 (7 + 7)
Cydnidae: Amaurocorinae	*Amaurocoris curtus* *	73	6	14 (7 + 7)	16 (9 + 7)	12 (6 + 6)	11 (7 + 4)	14 (7 + 7)
Cydnidae: Sehirinae	*Adomerus biguttatus* *	73	6	12 (6 + 6)	18 (10 + 8)	14 (7 + 7)	9 (6 + 3)	14 (7 + 7)
Parastrachiidae	*Parastrachia japonensis* *	73	6	12 (6 + 6)	18 (10 + 8)	14 (7 + 7)	9 (6 + 3)	14 (7 + 7)
Cydnidae: Garsauriinae	*Garsauria aradoides* *	74	6	17 (9 + 8)	11 (6 + 5)	15 (8 + 7)	10 (7 + 3)	15 (7 + 8)
Cydnidae: Amnestinae	*Amnestus zacki* *	73	6	11 (10 + 1)	13 (7 + 6)	12 (5 + 7)	10 (5 + 5)	21 (11 + 10)
Cydnidae: Cydninae	*Chilocoris piceus* **	81	7	14 (7 + 7)	16 (9 + 7)	15 (8 + 7)	11 (7 + 4)	18 (8 + 10)

## Data Availability

Data contained within the article.

## Supplementary Materials

The supporting information can be downloaded at: https://www.mdpi.com/article/10.3390/ijms25020939/s1.
