# Supplementary material for: Inconsistencies in the Classification of the Family Cydnidae (Hemiptera: Heteroptera: Pentatomoidea) Revealed by Molecular Apomorphies in the Secondary and Tertiary Structures of 18S rRNA Length-Variable Region L (LVR L)
Source: Int J Mol Sci. 2024 Jan 11;25(2):939. doi: 10.3390/ijms25020939 (PMC10815949; doi:10.3390/ijms25020939)
Supplement: Supplementary file 1 [file ijms-25-00939-s001.zip › Supplementary File S2.pdf]

File S2. Number of nucleotides in the LVR L subregions of the analysed species, with the assignment to the spermathecal types and facies recovered in the family Cydnidae and classified within the higher taxon (family, subfamily, tribe) of the superfamily Pentatomoidea.

Subregion	Number of nucleotides	Secondary structure	Species	Spermathecal type/facies	Higher taxon
L(A)	17 (9+8)	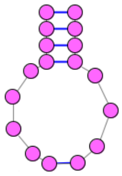 1	*Thaumastella elizabethae*	–	Thaumastellidae
			Garsauria aradoides	garsaurinae type	Cydnidae: Garsaurinae
	15 (8+7)	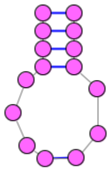 2	*Cyrtomenus emarginatus*<br>*Rhytidoporus indentatus*	‘cydnoid’ type / geotoman facies	Cydnidae:<br>Cydninae: Geotomini
	14 (7+7)	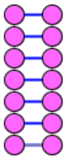 3	*Amaurocoris curtus*	amaurocorinae type	Cydnidae: Amaurocorinae
		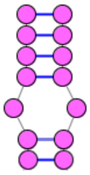 4	*Thyreocoris scarabaeoides*	–	Thyreocoridae
			Stibaropus indonesicus	‘cydnoid’ type / geotoman facies	Cydnidae: Cephalocteinae
			Fromundus pygmaeus<br>*Macroscytus badius*		Cydnidae: Cydninae: Geotomini
			Adrisa romani	‘cydnoid’ type / adrisan facies	
			Pseudoscoparipes fraterculus	‘cydnoid’ type / scoparipan facies	
			Cydnus aterrimus<br>*Chilocoris piceus*	‘cydnoid’ type / cydnan facies	Cydnidae: Cydninae: Cydnini
	12 (6+6)	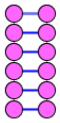 5	*Ochetostethomorpha secunda*	‘cydnoid’ type / ochetostethan facies	Cydnidae: Sehirinae: Sehirini
			Adomerus biguttatus	‘cydnoid’ type / sehiran facies	Cydnidae: Sehirinae: Sehirini
			Parastrachia japonensis	–	Parastrachiidae
	11 (10+1)	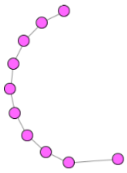 6	*Amnestus zacki*	amnestinae type	Cydnidae: Amnestinae

File S2. Number of nucleotides in the LVR L subregions of the analysed species, with the assignment to the spermathecal types and facies recovered in the family Cydnidae and classified within the higher taxon (family, subfamily, tribe) of the superfamily Pentatomoidea.

L(B)	19 (11+8)	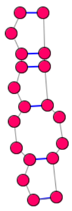 **1**	*Macroscytus badius*	'cydnoid' type / geotoman facies	Cydnidae: Cydninae: Geotomini
	18 (10+8)	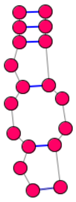 **2**	*Cyrtomenus emarginatus*<br>*Rhytidoporus indentatus*	'cydnoid' type / geotoman facies	Cydnidae: Cydninae: Geotomini
		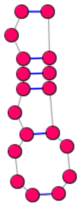 **3**	*Adomerus biguttatus*	'cydnoid' type / sehiran facies	Cydnidae: Sehirinae: Sehirini
			Parastrachia japonensis	–	Parastrachiidae
		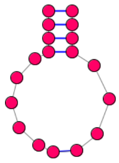 **4**	*Ochetostethomorpha secunda*	'cydnoid' type / ochetostethan facies	Cydnidae: Sehirinae: Sehirini
	17 (10+7)	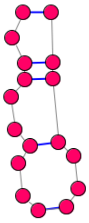 **5**	*Thyreocoris scarabaeoides*	–	Thyreocoridae
	16 (9+7)	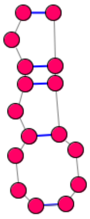 **6**	*Fromundus pygmaeus*	'cydnoid' type / geotoman facies	Cydnidae: Cydninae: Geotomini
			Adrisa romani	'cydnoid' type / adrisan facies	
			Pseudoscoparipes fraterculus	'cydnoid' type / scoparipan facies	
			Cydnus aterrimus	'cydnoid' type / cydnan facies	Cydnidae: Cydninae: Cydnini

File S2. Number of nucleotides in the LVR L subregions of the analysed species, with the assignment to the spermathecal types and facies recovered in the family Cydnidae and classified within the higher taxon (family, subfamily, tribe) of the superfamily Pentatomoidea.

L(B)	16 (9+7)	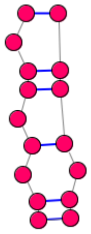 **7**	*Stibaropus indonesicus*	‘cydnoid’ type / geotoman facies	Cydnidae: Cephalocteinae
		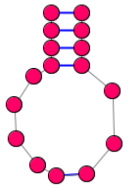 **8**	*Chilocoris piceus*	‘cydnoid’ type / cydnan facies	Cydnidae: Cydninae: Cydnini
		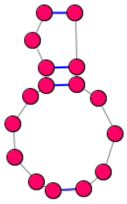 **9**	*Amaurocoris curtus*	amaurocorinae type	Cydnidae: Amaurocorinae
	13 (7+6)	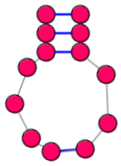 **10**	*Amnestus zacki*	amnestinae type	Cydnidae: Amnestinae
	11 (6+5)	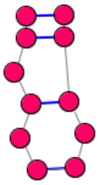 **11**	*Garsauria aradoides*	garsaurinae type	Cydnidae: Garsaurinae
			Thaumastella elizabethae	–	Thaumastellidae
L(C)	16 (8+8)	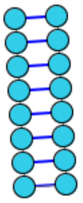 **1**	*Thaumastella elizabethae*	–	Thaumastellidae
	15 (8+7)	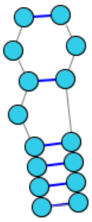 **2**	*Stibaropus indonesicus*	‘cydnoid’ type / geotoman facies	Cydnidae: Cephalocteinae

File S2. Number of nucleotides in the LVR L subregions of the analysed species, with the assignment to the spermathecal types and facies recovered in the family Cydnidae and classified within the higher taxon (family, subfamily, tribe) of the superfamily Pentatomoidea.

L(C)	15 (8+7)	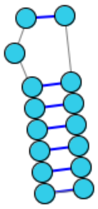 3	*Fromundus pygmaeus*	'cydnoid' type / geotoman facies	Cydnidae: Cydninae: Geotomini
		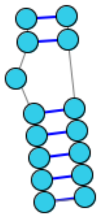 4	*Garsauria aradoides*	garsaurinae type	Cydnidae: Garsaurinae
		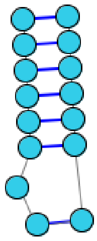 5	*Chilocoris piceus*	'cydnoid' type / cydnan facies	Cydnidae: Cydninae: Cydnini
	14 (7+7)	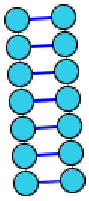 6	*Adrisa romani*	'cydnoid' type / adrisan facies	Cydnidae: Cydninae: Geotomini
			Pseudoscoparipes fraterculus	'cydnoid' type / scoparipan facies	
			Adomerus buguttatus	'cydnoid' type / sehiran facies	Cydnidae: Sehirinae: Sehirini
			Parastrachia japonensis	–	Parastrachiidae
			Thyreocoris scarabaeoides	–	Thyreocoridae
		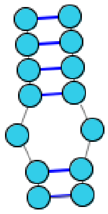 7	*Ochetostethomorpha secunda*	'cydnoid' type / ochetostethan facies	Cydnidae: Sehirinae: Sehirini
	12 (6+6)	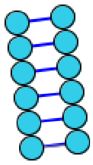 8	*Cyrtomenus emarginatus*<br>*Macroscytus badius*	'cydnoid' type / geotoman facies	Cydnidae: Cydninae: Geotomini
			Amaurocoris curtus	amaurocorinae type	Cydnidae: Amaurocorinae

File S2. Number of nucleotides in the LVR L subregions of the analysed species, with the assignment to the spermathecal types and facies recovered in the family Cydnidae and classified within the higher taxon (family, subfamily, tribe) of the superfamily Pentatomoidea.

LC	12 (5+7)	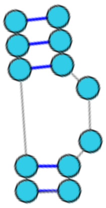 **9**	*Amnestus zacki*	amnestinae type	Cydnidae: Amnestinae
	10 (5+5)	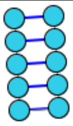 **10**	*Rhytidoporus indentatus*	'cydnoid' type / geotoman facies	Cydnidae: Cydninae: Geotomini
			Cydnus aterrimus	'cydnoid' type / cydnan facies	Cydnidae: Cydninae: Cydnini
L(D)	11 (7+4)	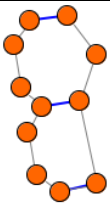 **1**	*Rhytidoporus indentatus*	'cydnoid' type / geotoman facies	Cydnidae: Cydninae: Geotomini
	11 (7+4)	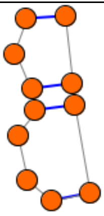 **2**	*Amaurocoris curtus*	amaurocorinae type	Cydnidae: Amaurocorinae
			Chilocoris piceus	'cydnoid' type / cydnan facies	Cydnidae: Cydninae: Cydnini
	10 (7+3)	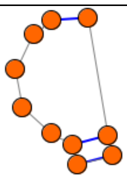 **3**	*Garsauria aradoides*	garsaurinae type	Cydnidae: Garsaurinae
			Cydnus aterrimus	'cydnoid' type / cydnan facies	Cydnidae: Cydninae: Cydnini
	10 (5+5)	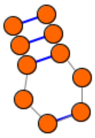 **4**	*Amnestus zacki*	amnestinae type	Cydnidae: Amnestinae
	9 (6+3)	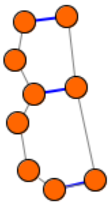 **5**	*Stibaropus indonesicus*	'cydnoid' type / geotoman facies	Cydnidae: Cephalocteinae
			Fromundus pygmaeus		Cydnidae: Cydninae: Geotomini
			Macroscytus badius		
			Cyrtomenus emarginatus		
			Adrisa romani	'cydnoid' type / adrisan facies	
			Pseudoscoparipes fraterculus	'cydnoid' type / scoparipan facies	
			Ochetostethomorpha secunda	'cydnoid' type / ochetostethan facies	Cydnidae: Sehirinae: Sehirini
			Adomerus biguttatus	'cydnoid' type / sehiran facies	
			Parastrachia japonensis	–	Parastrachiidae
			Thyreocoris scarabaeoides	–	Thyreocoridae

File S2. Number of nucleotides in the LVR L subregions of the analysed species, with the assignment to the spermathecal types and facies recovered in the family Cydnidae and classified within the higher taxon (family, subfamily, tribe) of the superfamily Pentatomoidea.

L(D)	8 (5+3)	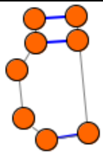 6	*Thaumastella elizabethae*	–	Thaumastellidae
L(E)	21 (11+10)	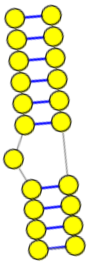 1	*Amnestus zacki*	amnestinae type	Cydnidae: Amnestinae
	20 (10+10)	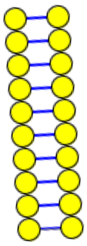 2	*Thaumastella elizabethae*	–	Thaumastellidae
	19 (9+10)	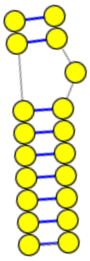 3	*Cydnus aterrimus*	'cydnoid' type / cydnan facies	Cydnidae: Cydninae: Cydnini
	18 (8+10)	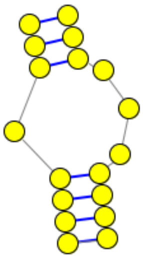 4	*Chilocoris piceus*	'cydnoid' type / cydnan facies	Cydnidae: Cydninae: Cydnini
	16 (8+8)	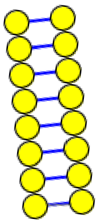 5	*Stibaropus indonesicus*	'cydnoid' type / geotoman facies	Cydnidae: Cephalocteinae
			Fromundus pygmaeus		Cydnidae: Cydninae: Geotomini
			Macroscytus badius		
			Cyrtomenus emarginatus		
			Adrisa romani	'cydnoid' type / adrisan facies	
			Pseudoscoparipes fraterculus	'cydnoid' type / scoparipan facies	
			Ochetostethomorpha secunda	'cydnoid' type / ochetostethan facies	Cydnidae: Sehirinae: Sehirini

File S2. Number of nucleotides in the LVR L subregions of the analysed species, with the assignment to the spermathecal types and facies recovered in the family Cydnidae and classified within the higher taxon (family, subfamily, tribe) of the superfamily Pentatomoidea.

L(E)	15 (7+8)	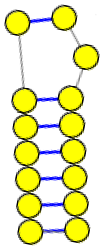 6	*Garsauria aradoides*	garsaurinae type	Cydnidae: Garsaurinae
	14 (7+7)	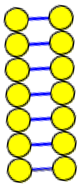 7	*Amaurocoris curtus*	amaurocorinae type	Cydnidae: Amaurocorinae
			Adomerus biguttatus	'cydnoid' type / sehiran facies	Cydnidae: Sehirinae: Sehirini
			Parastrachia japonensis	–	Parastrachiidae
			Thyreocoris scarabaeoides	–	Thyreocoridae
L2	3	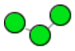 1	*Thaumastella elizabethae*	–	Thaumastellidae
L2	4	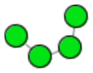 2	*Stibaropus indonesicus*	'cydnoid' type / geotoman facies	Cydnidae: Cephalocteinae
			Fromundus pygmaeus		Cydnidae: Cydninae: Geotomini
			Macroscytus badius		
			Cyrtomenus emarginatus		
			Rhytidoporus indentatus		
			Adrisa romani	'cydnoid' type / adrisan facies	
			Pseudoscoparipes fraterculus	'cydnoid' type / scoparipan facies	
			Cydnus aterrimus	'cydnoid' type / cydnan facies	Cydnidae: Cydninae: Cydnini
			Ochetostthomorpha secunda	'cydnoid' type / ochetostethan facies	Cydnidae: Sehirinae: Sehirini
	6	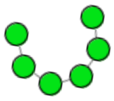 3	*Amnestus zacki*	amnestinae type	Cydnidae: Amnestinae
			Garsauria aradoides	garsaurinae type	Cydnidae: Garsaurinae
			Amaurocoris curtus	amaurocorinae type	Cydnidae: Amaurocorinae
			Parastrachia japonensis	–	Parastrachiidae
			Adomerus biguttatus	'cydnoid' type / sehiran facies	Cydnidae: Sehirinae: Sehirini
			Thyreocoris scarabaeoides	–	Thyreocoridae
	7	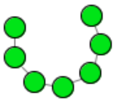 4	*Chilocoris piceus*	'cydnoid' type / cydnan facies	Cydnidae: Cydninae: Cydnini
