# Supplementary material for: Inconsistencies in the Classification of the Family Cydnidae (Hemiptera: Heteroptera: Pentatomoidea) Revealed by Molecular Apomorphies in the Secondary and Tertiary Structures of 18S rRNA Length-Variable Region L (LVR L)
Source: Int J Mol Sci. 2024 Jan 11;25(2):939. doi: 10.3390/ijms25020939 (PMC10815949; doi:10.3390/ijms25020939)

Global Quality Analysis (16)

Model	RMSD	Cutoff sphere 12Å	Cutoff sphere 16Å	INF WC	INF nWC	INF stacking	INF all	P-value	DI all	Clash Score	Deformation Proile
Adomerus.pdb	6.82	29%	9%	0.00	0.00	0.00	0.00	0.96	0.00	9.45	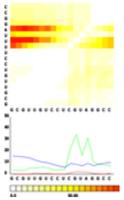
Adrisa.pdb	7.73	25%	4%	0.00	0.00	0.00	0.00	0.98	0.00	18.40	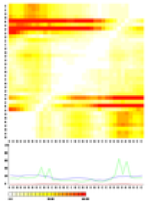
Amaurocoris.pdb	11.33	25%	2%	0.00	0.00	0.00	0.00	1.00	0.00	13.74	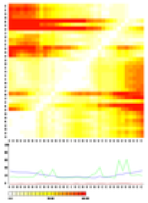
Amnestus.pdb	15.12	13%	2%	0.00	0.00	0.00	0.00	1.00	0.00	18.33	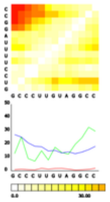
Chilocoris.pdb	11.73	13%	1%	0.00	0.00	0.00	0.00	1.00	0.00	19.22	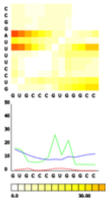

Global Quality Analysis (16)

Model	RMSD	Cutoff sphere 12Å	Cutoff sphere 16Å	INF WC	INF nWC	INF stacking	INF all	P-value	DI all	Clash Score	Deformation Proile
Cydnus.pdb	8.47	17%	4%	0.00	0.00	0.00	0.00	0.99	0.00	16.71	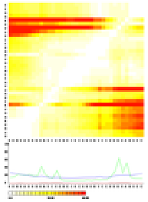
Cyrtomenus.pdb	5.88	42%	2%	0.00	0.00	0.00	0.00	0.73	0.00	15.20	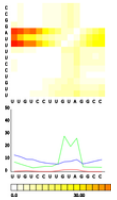
Fromundus.pdb	6.58	42%	14%	0.03	0.00	0.00	0.03	0.84	262.59	18.57	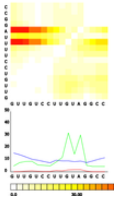
Garsauria.pdb	6.85	41%	12%	0.00	0.09	0.00	0.02	0.80	294.46	14.31	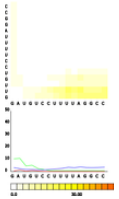
Macroscytus.pdb	6.46	37%	2%	0.03	0.00	0.00	0.02	0.83	274.58	18.59	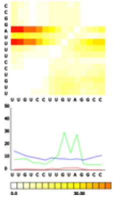

Global Quality Analysis (16)

Model	RMSD	Cutoff sphere 12Å	Cutoff sphere 16Å	INF WC	INF nWC	INF stacking	INF all	P-value	DI all	Clash Score	Deformation Proile
Ochetostethomorpha.pdb	9.81	30%	13%	0.00	0.00	0.00	0.00	1.00	0.00	12.45	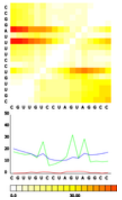
Parastrachia.pdb	7.17	30%	9%	0.00	0.00	0.20	0.02	0.97	308.20	17.62	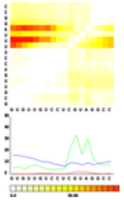
Pseudoscoparipe.pdb	6.70	29%	6%	0.00	0.00	0.00	0.00	0.96	0.00	19.70	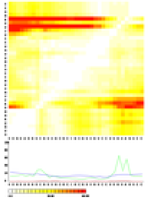
Rhytidoporus.pdb	5.47	49%	13%	0.00	0.00	0.00	0.00	0.71	0.00	17.34	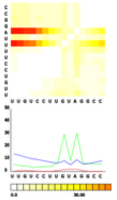
Stibaropus.pdb	6.49	42%	12%	0.03	0.00	0.00	0.03	0.87	251.65	19.41	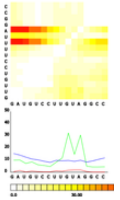

Global Quality Analysis (16)

Model	RMSD	Cutoff sphere 12Å	Cutoff sphere 16Å	INF WC	INF nWC	INF stacking	INF all	P-value	DI all	Clash Score	Deformation Proile
Thyreocoris.pdb	6.28	29%	5%	0.04	0.00	0.00	0.02	0.84	260.35	19.86	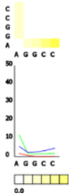

Averaged

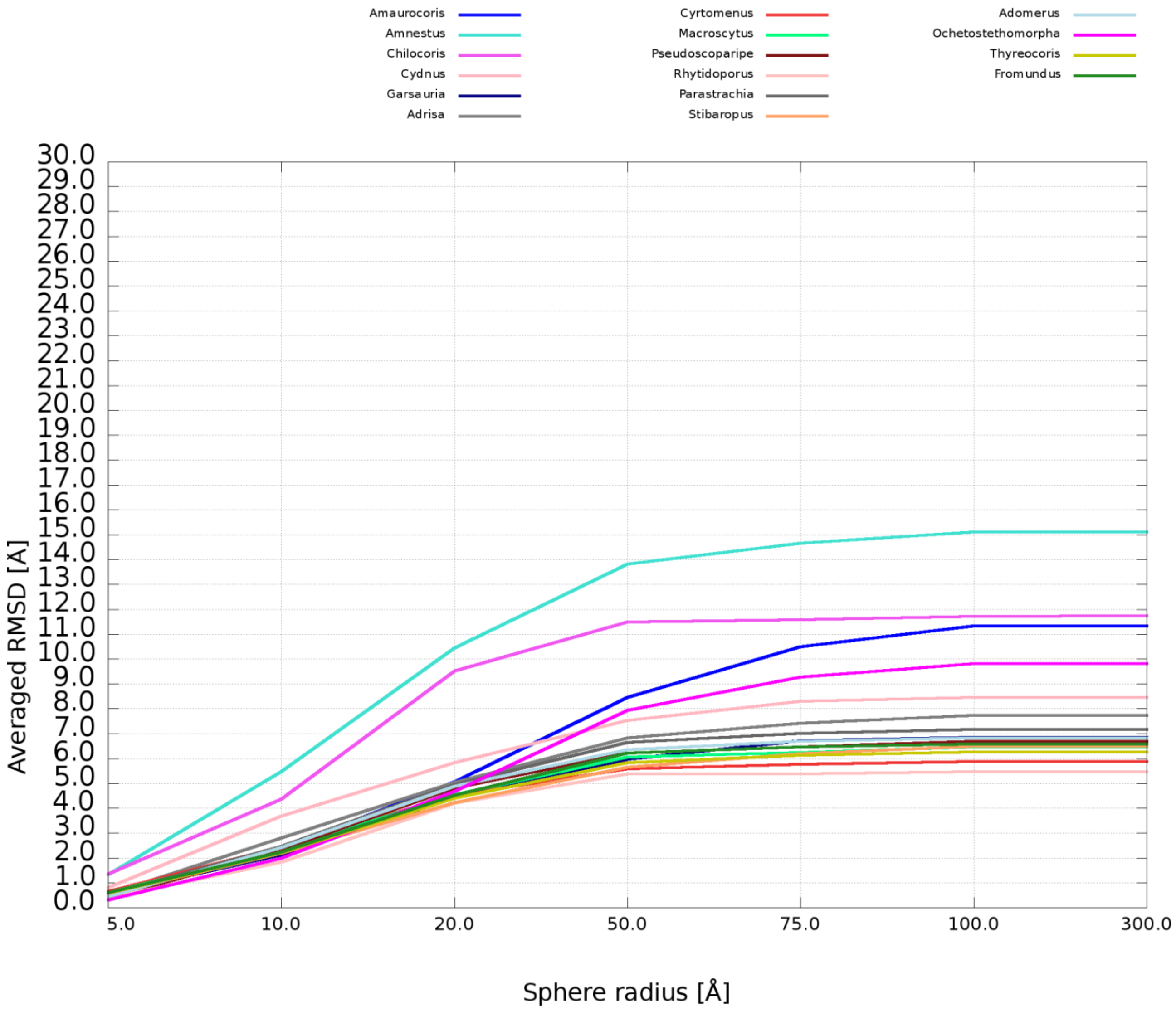

Amaurocoris
Amnestus
Chilocoris
Cydnus
Garsauria
Adrisa
Cyrtomenus
Macroscytus
Pseudoscoparipe
Rhytidoporus
Parastrachia
Stibaropus
Adomerus
Ochetostethomorpha
Thyreocoris
Fromundus
Averaged RMSD [Å]
30.0
29.0
28.0
27.0
26.0
25.0
24.0
23.0
22.0
21.0
20.0
19.0
18.0
17.0
16.0
15.0
14.0
13.0
12.0
11.0
10.0
9.0
8.0
7.0
6.0
5.0
4.0
3.0
2.0
1.0
0.0
5.0
10.0
20.0
50.0
75.0
100.0
300.0
Sphere radius [Å]

Cutoff 2Å

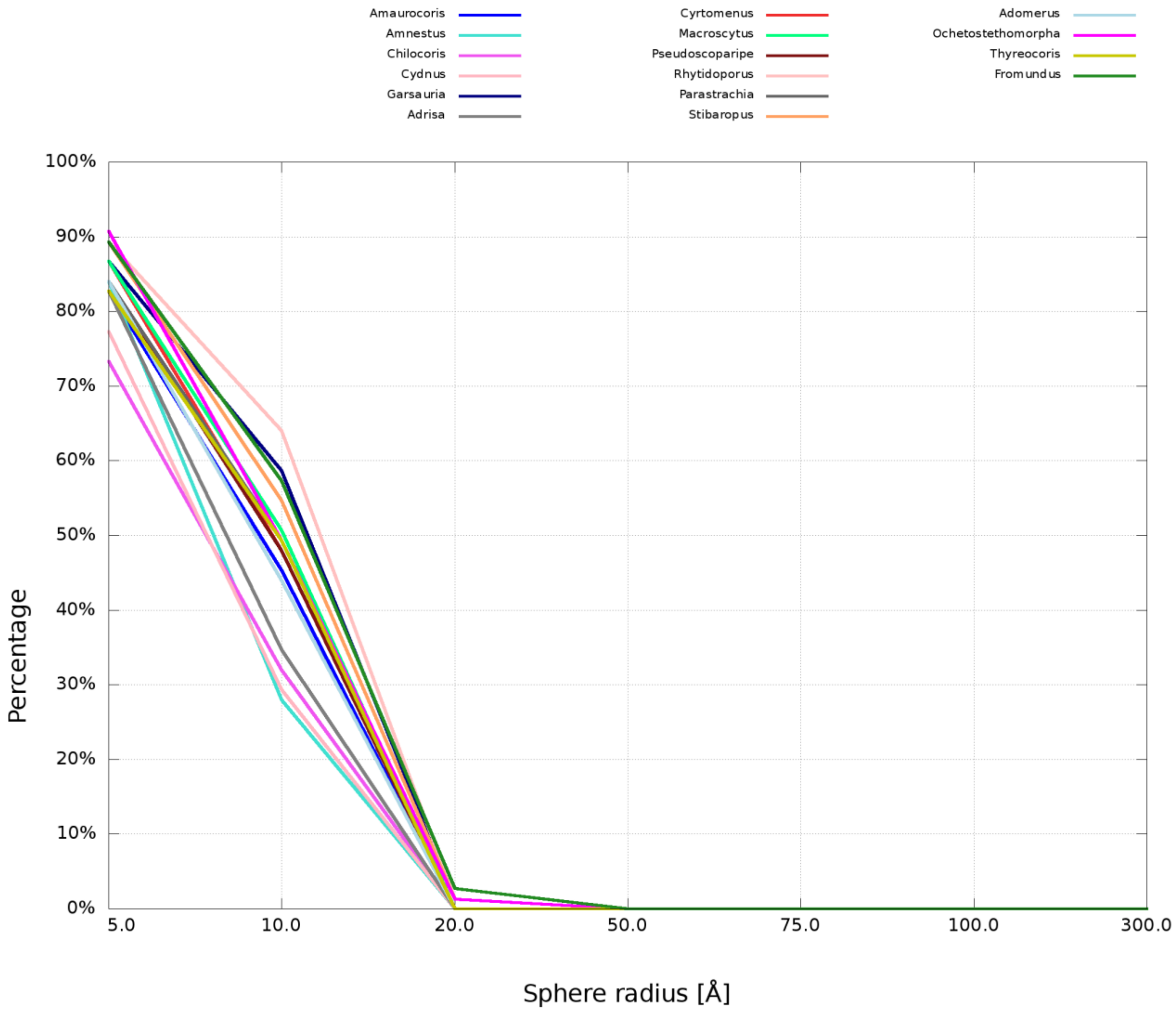

Amaurocoris
Amnestus
Chilocoris
Cydnus
Garsauria
Adrisa
Cyrtomenus
Macroscytus
Pseudoscoparipe
Rhytidoporus
Parastrachia
Stibaropus
Adomerus
Ochetostethomorpha
Thyreocoris
Fromundus
100%
90%
80%
70%
60%
50%
40%
30%
20%
10%
0%
Percentage
5.0
10.0
20.0
50.0
75.0
100.0
300.0
Sphere radius [Å]

Multi-model for 5Å

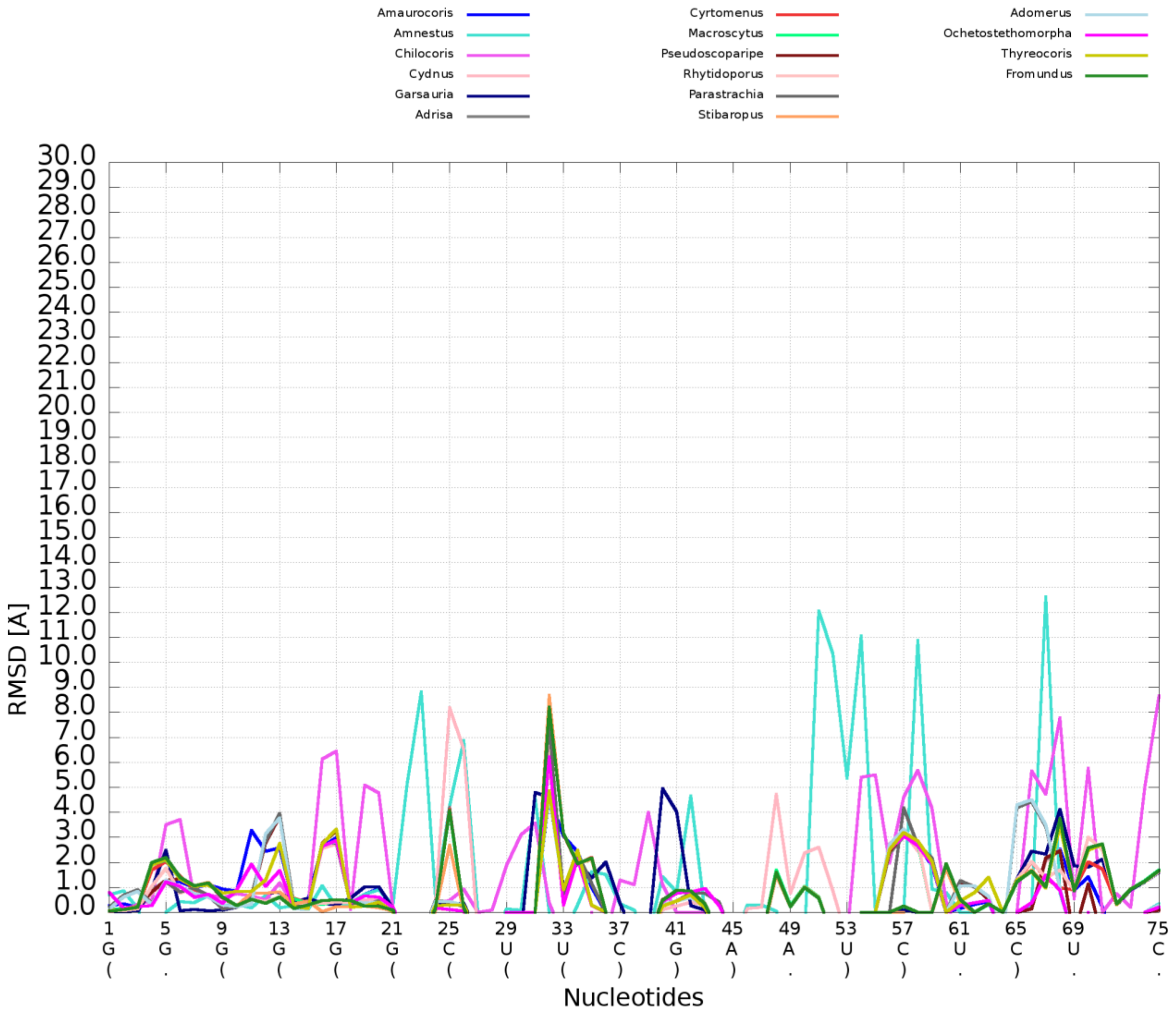

Multi-model for 10Å

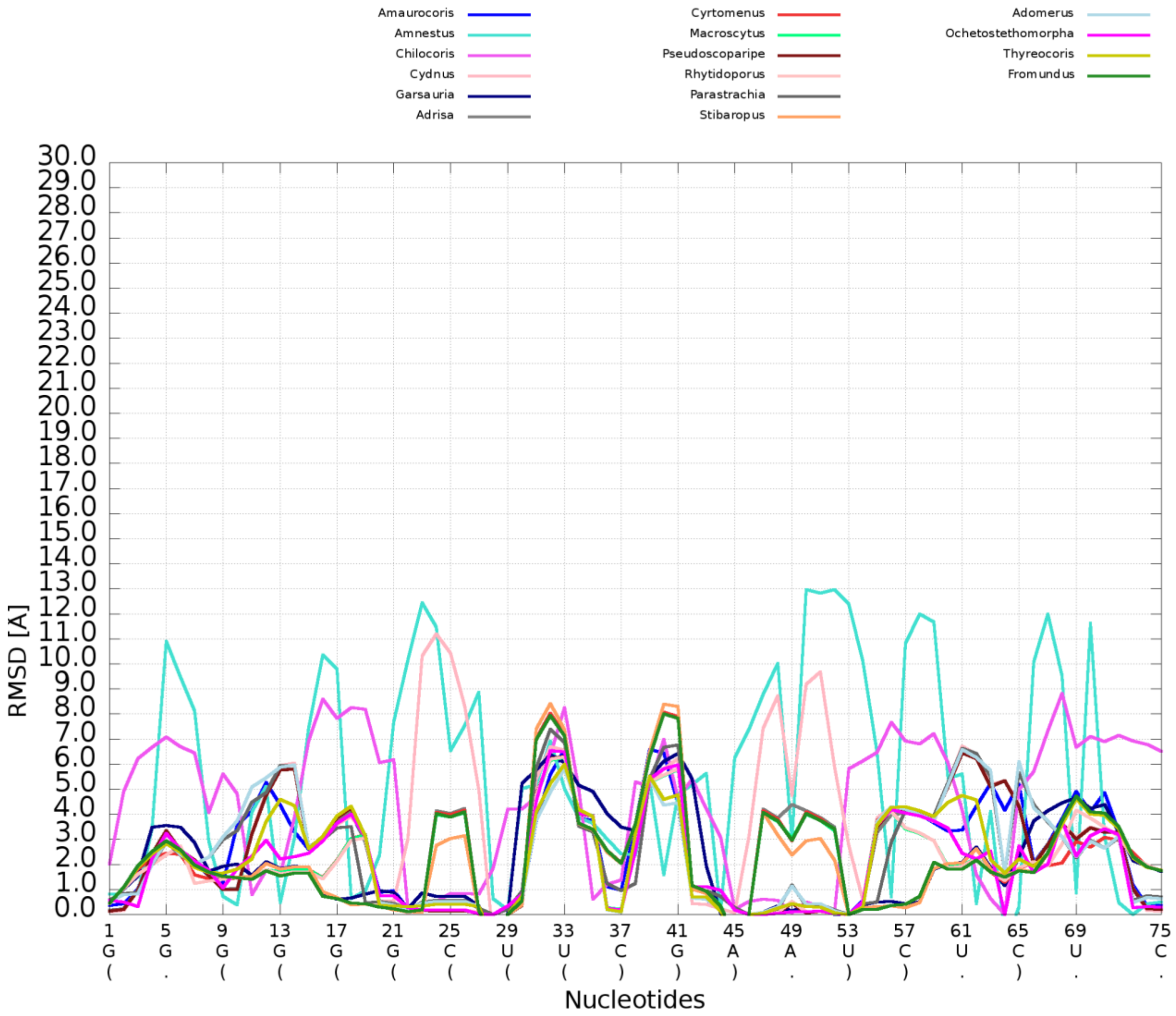

Multi-model for 20Å

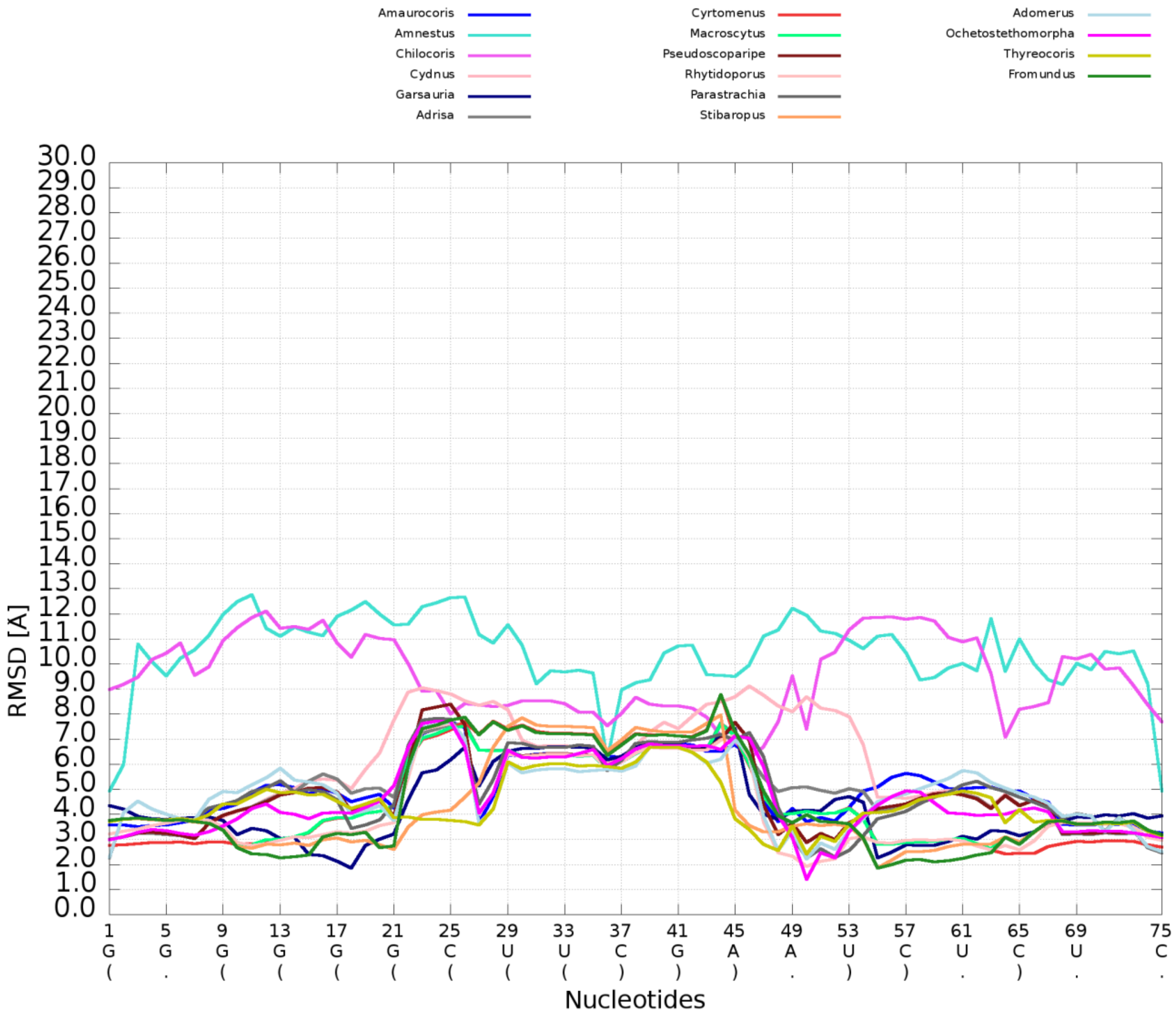

Multi-model for 50Å

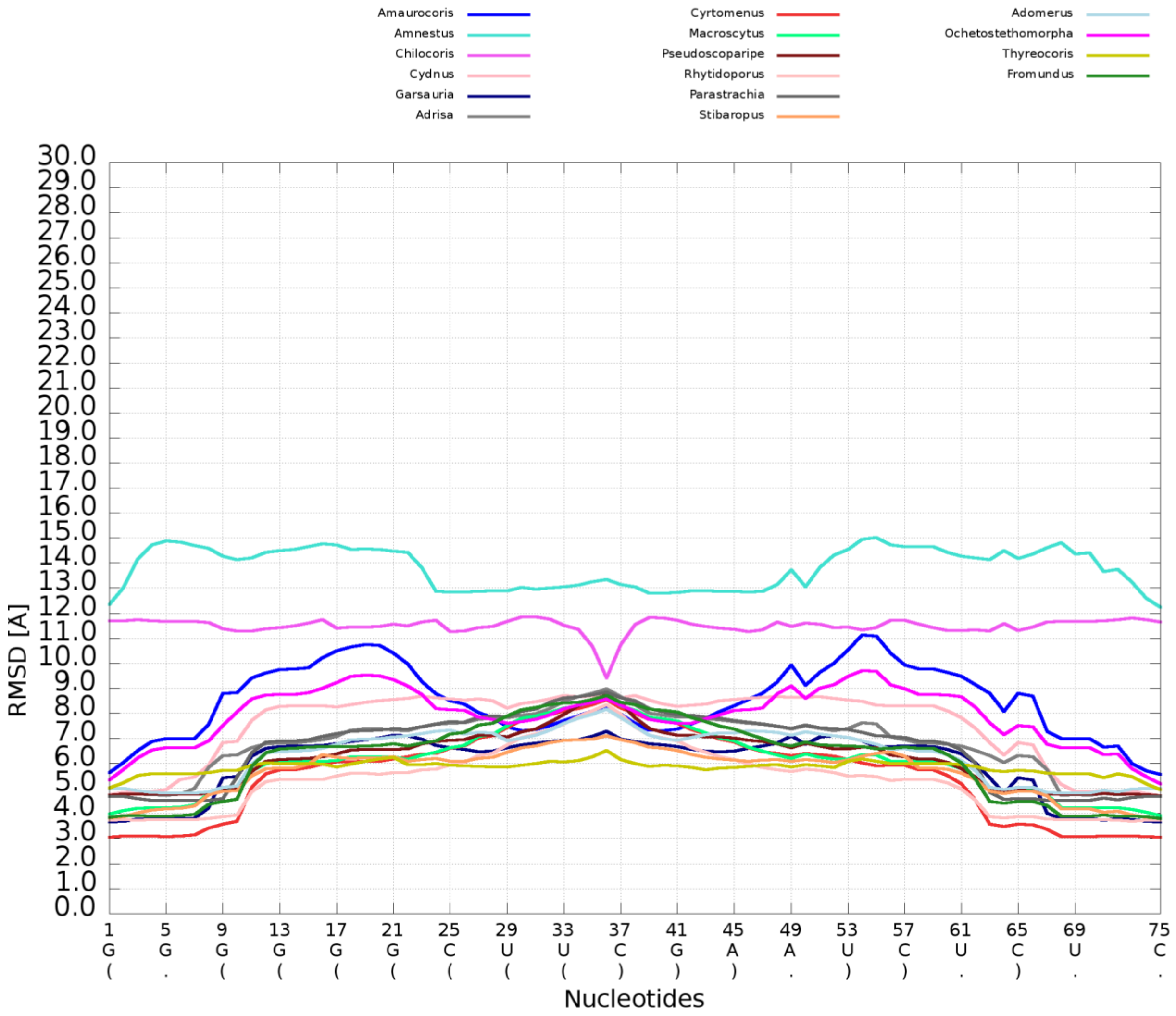

Multi-model for 75Å

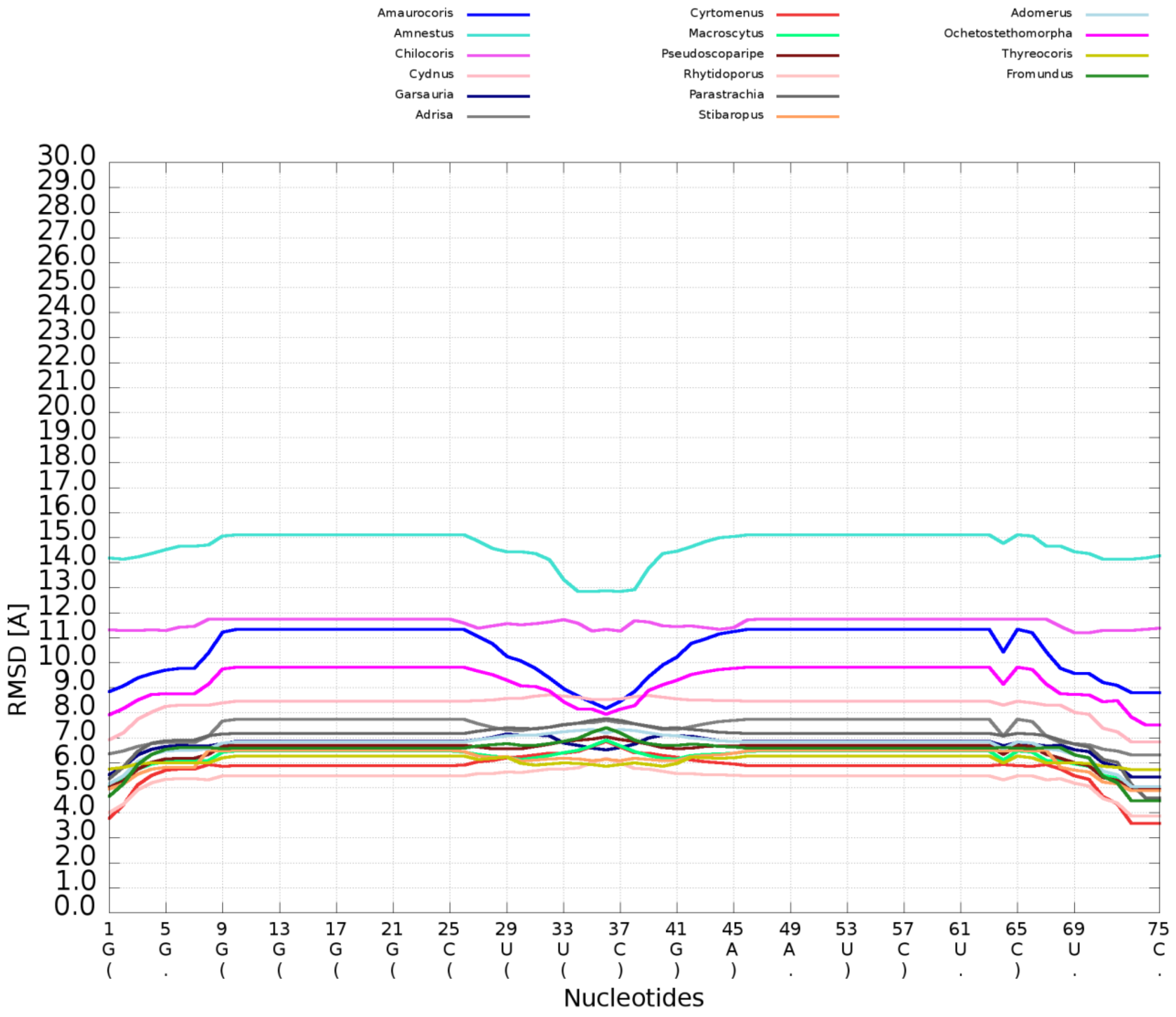

Multi-model for 100Å

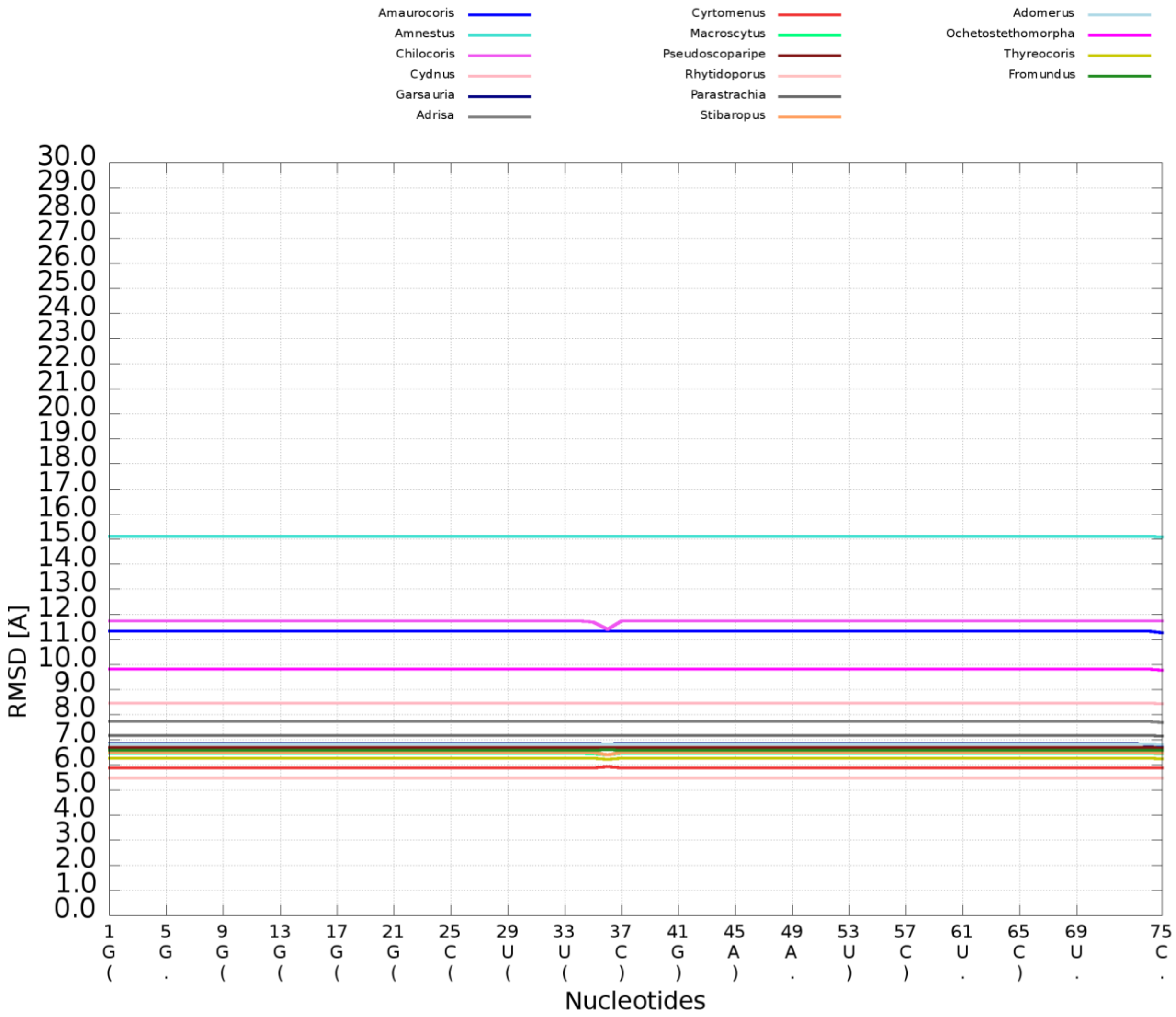

Models (16)

Model	2D map	3D plot
Adomerus.pdb	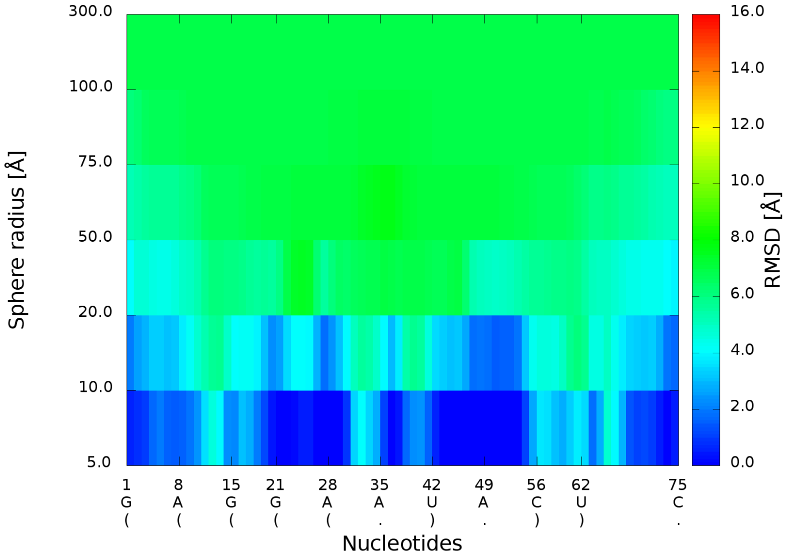	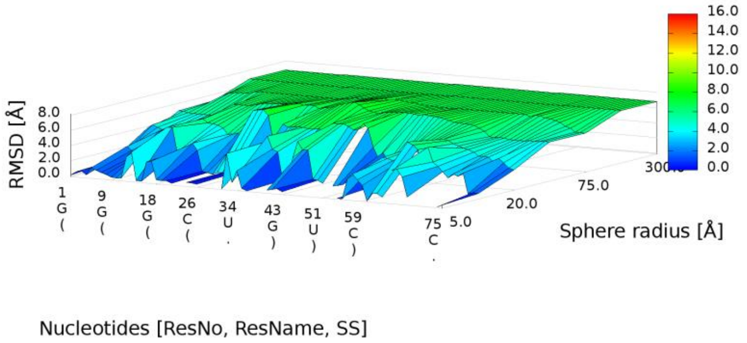

Adrisa.pdb

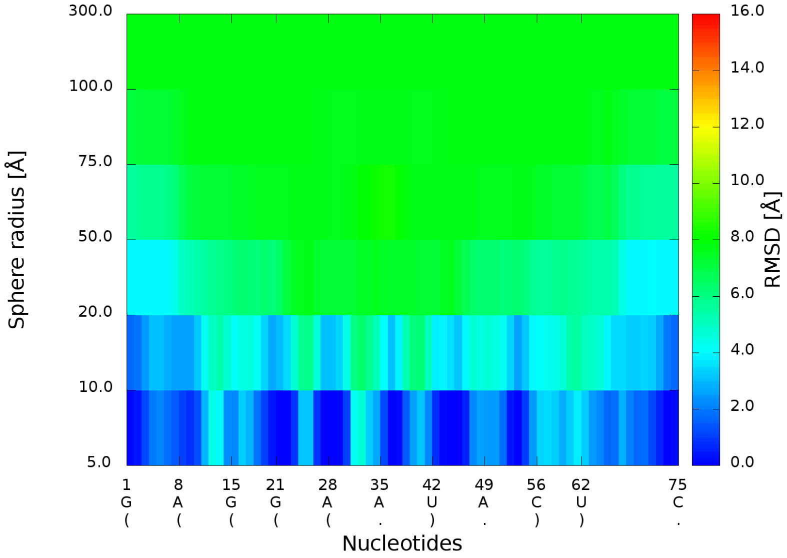

Sphere radius [Å]
300.0
100.0
75.0
50.0
20.0
10.0
5.0
RMSD [Å]
16.0
14.0
12.0
10.0
8.0
6.0
4.0
2.0
0.0
Nucleotides

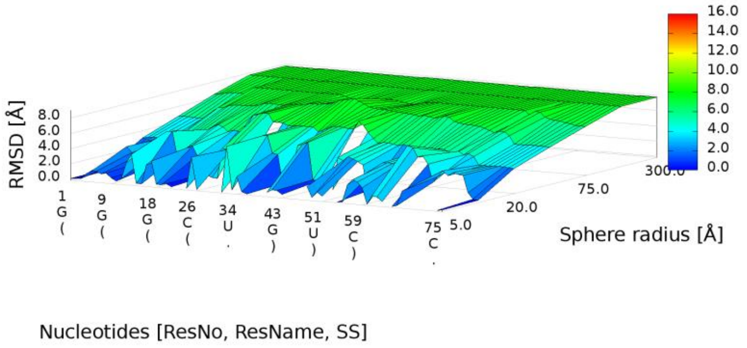

RMSD [Å]
Sphere radius [Å]
Nucleotides [ResNo, ResName, SS]

Amaurocoris.pdb

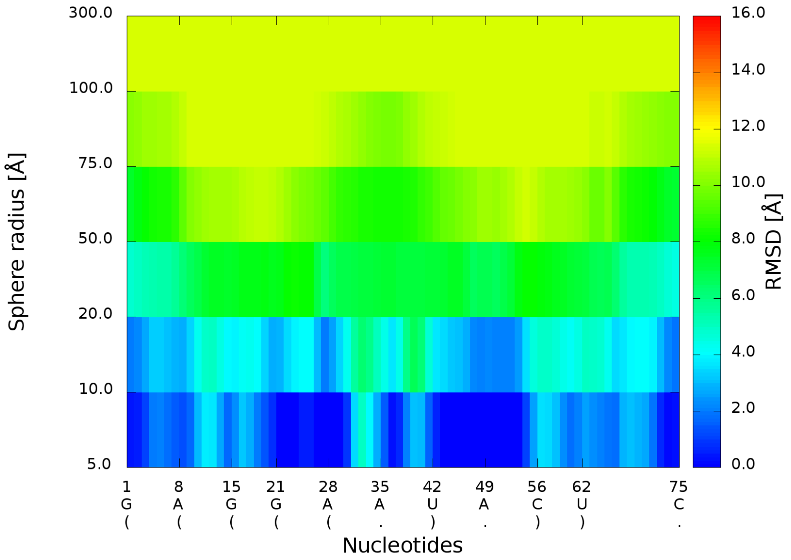

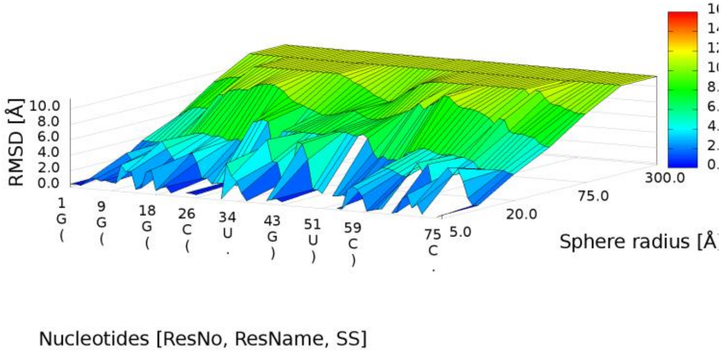

Amnestus.pdb

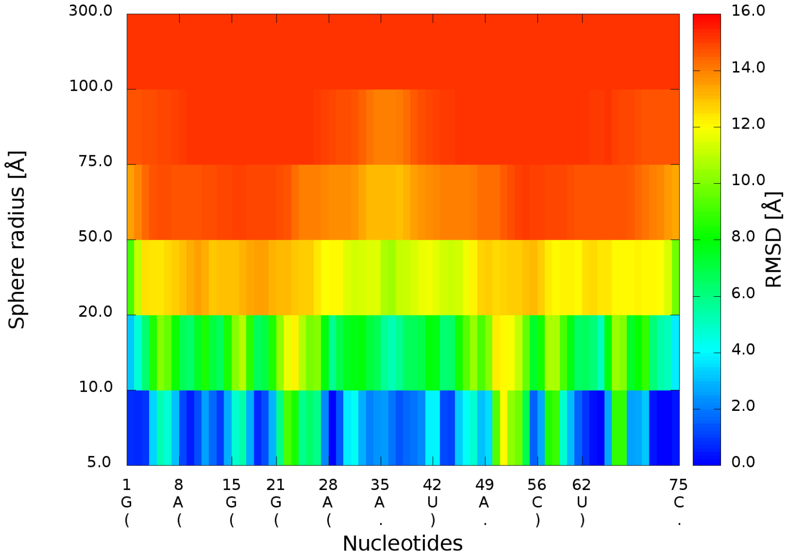

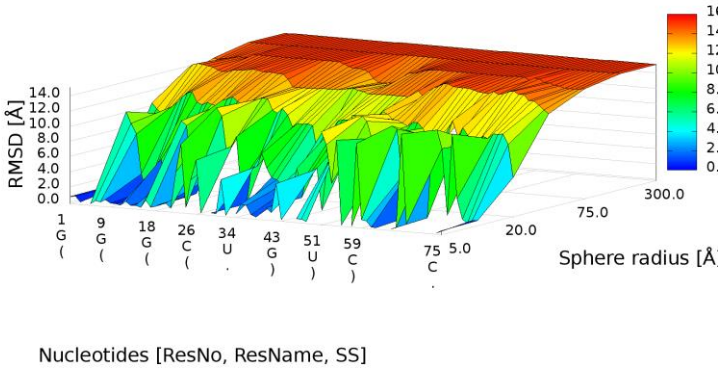

Chilocoris.pdb

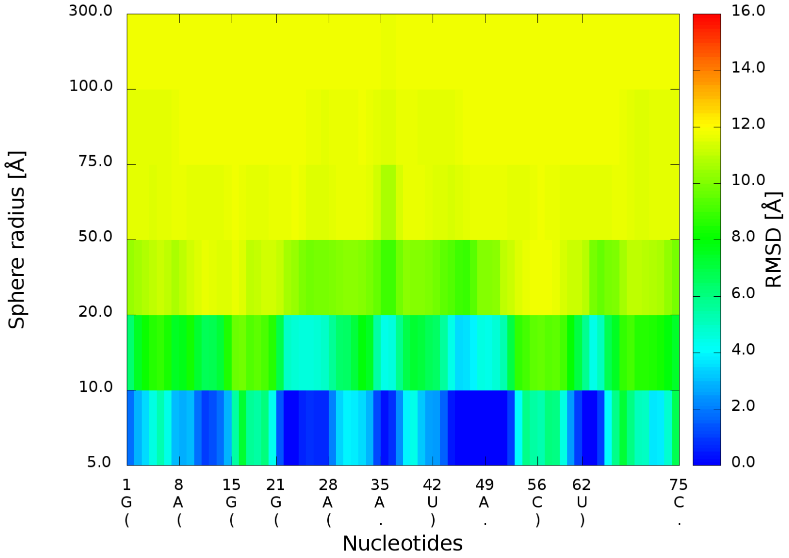

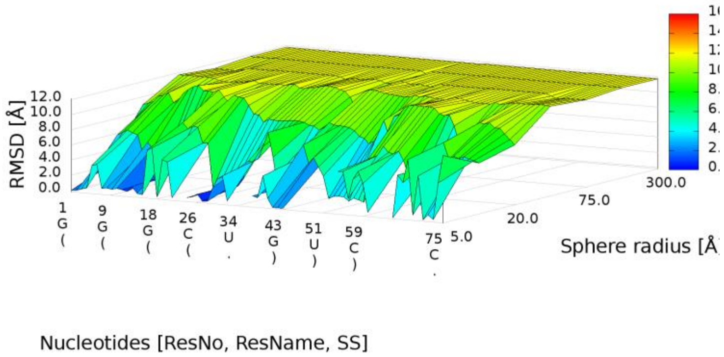

Cydnus.pdb

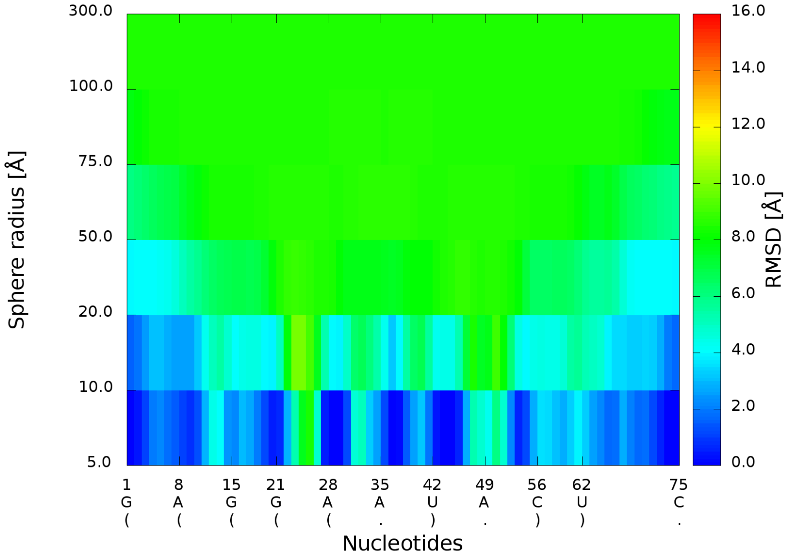

300.0
100.0
75.0
50.0
20.0
10.0
5.0
Sphere radius [Å]
16.0
14.0
12.0
10.0
8.0
6.0
4.0
2.0
0.0
RMSD [Å]
1 G (
8 A (
15 G (
21 G (
28 A (
35 A .
42 U)
49 A .
56 C)
62 U)
75 C .
Nucleotides

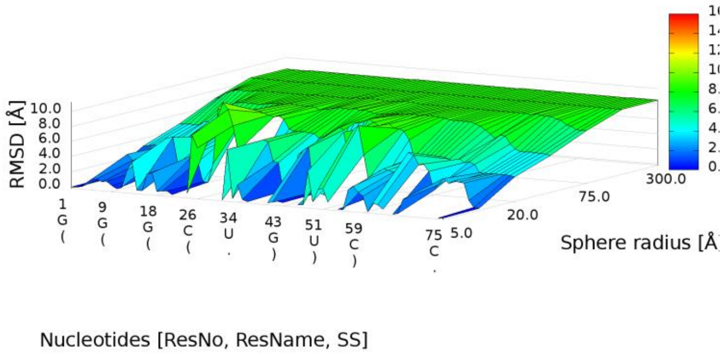

RMSD [Å]
10.0
8.0
6.0
4.0
2.0
0.0
1 G (
9 G (
18 G (
26 C (
34 U .
43 G)
51 U)
59 C)
75 C .
5.0
20.0
75.0
300.0
Nucleotides [ResNo, ResName, SS]

Cyrtomenus.pdb

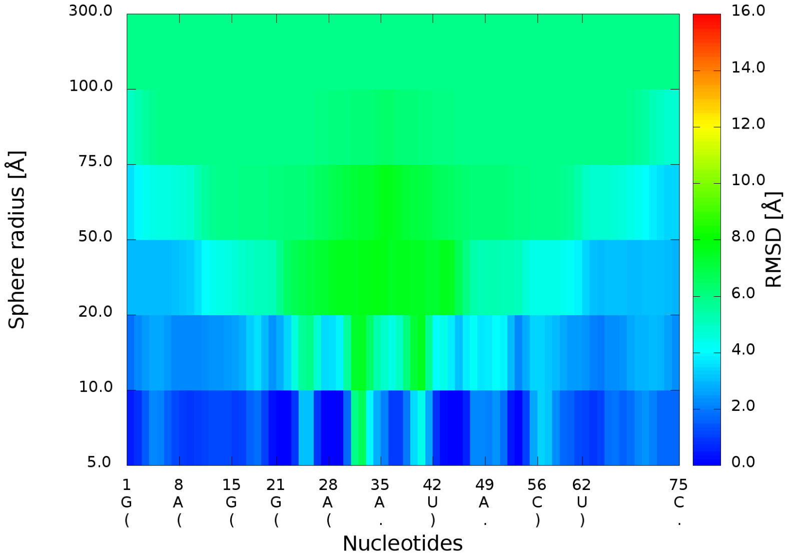

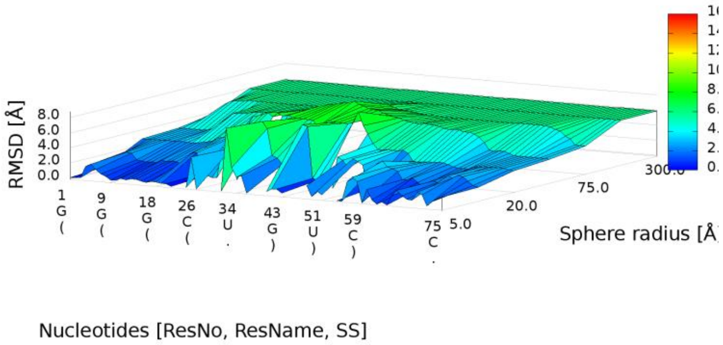

Fromundus.pdb

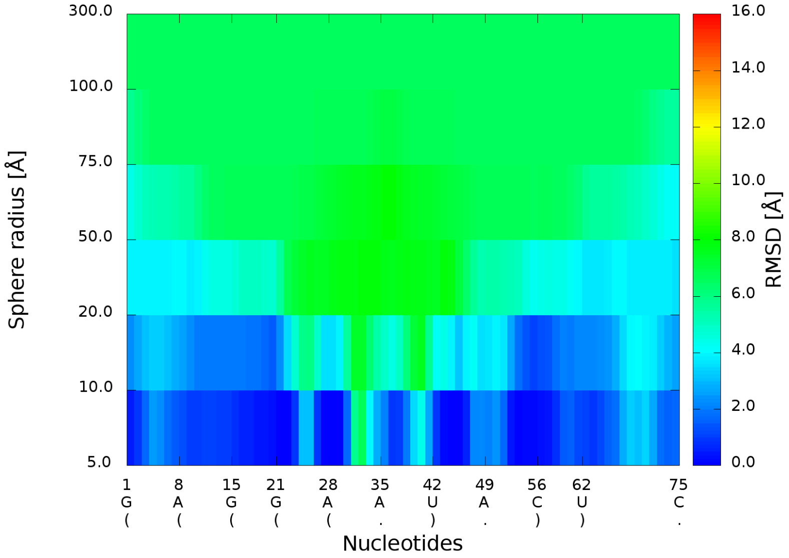

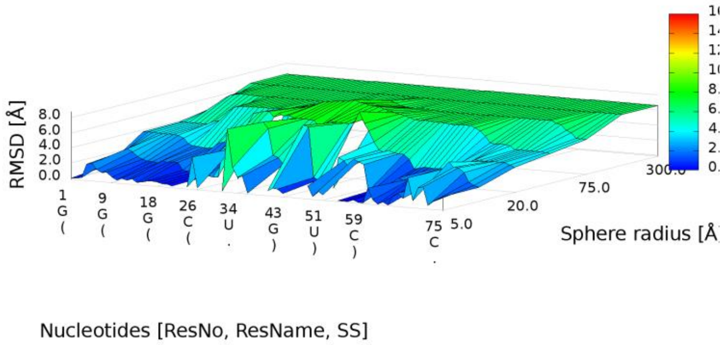

Garsauria.pdb

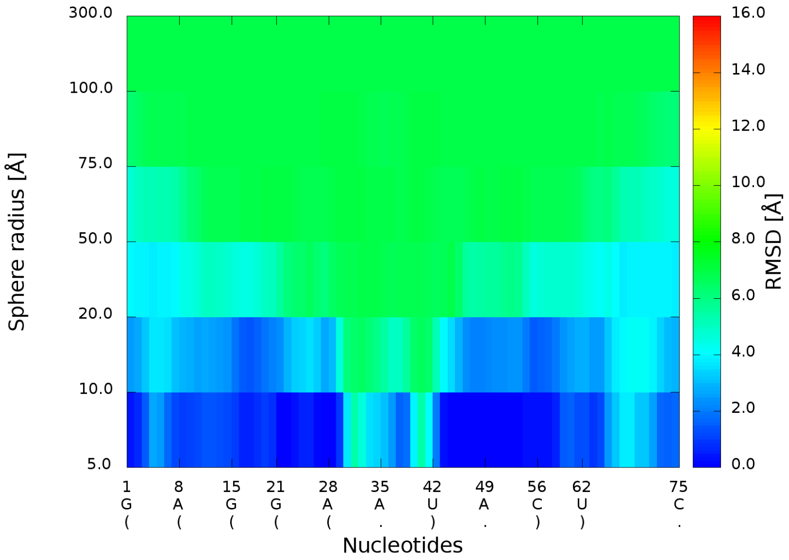

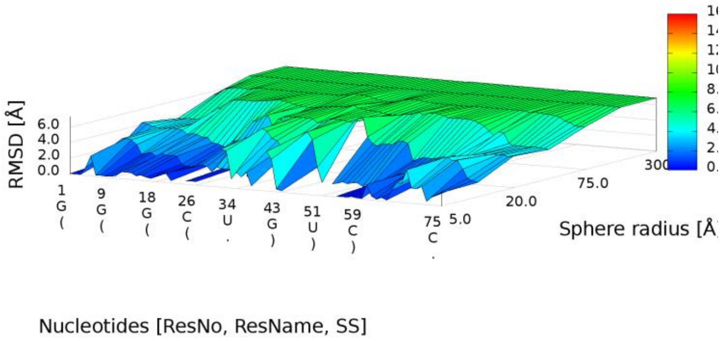

Macroscytus.pdb

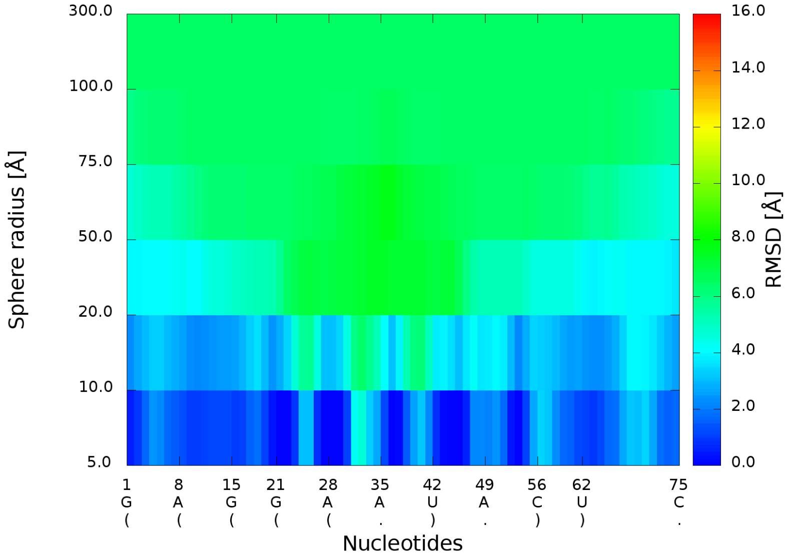

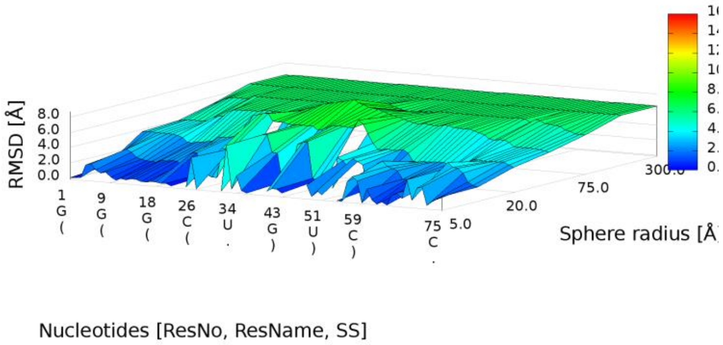

Ochetostethomorpha.pdb

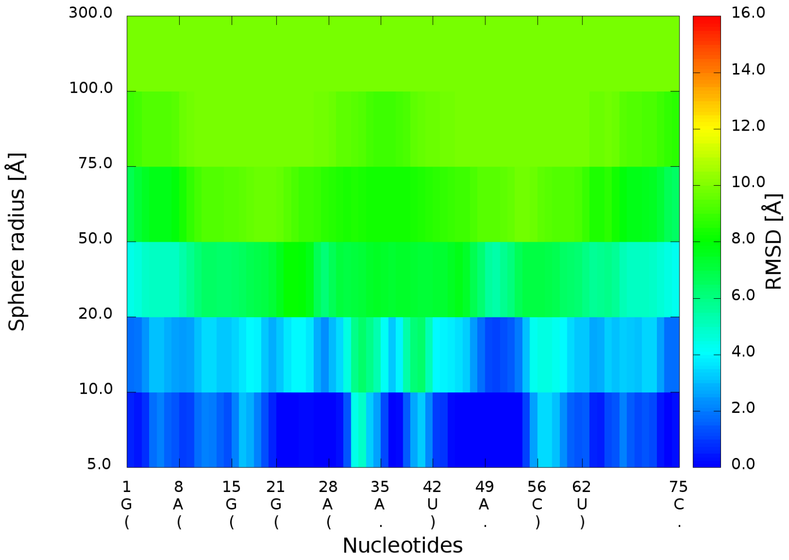

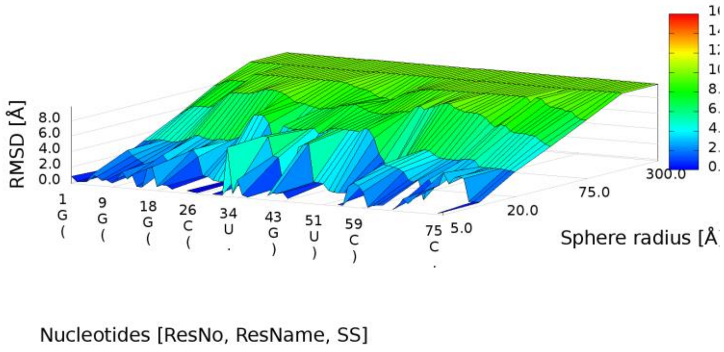

Parastrachia.pdb

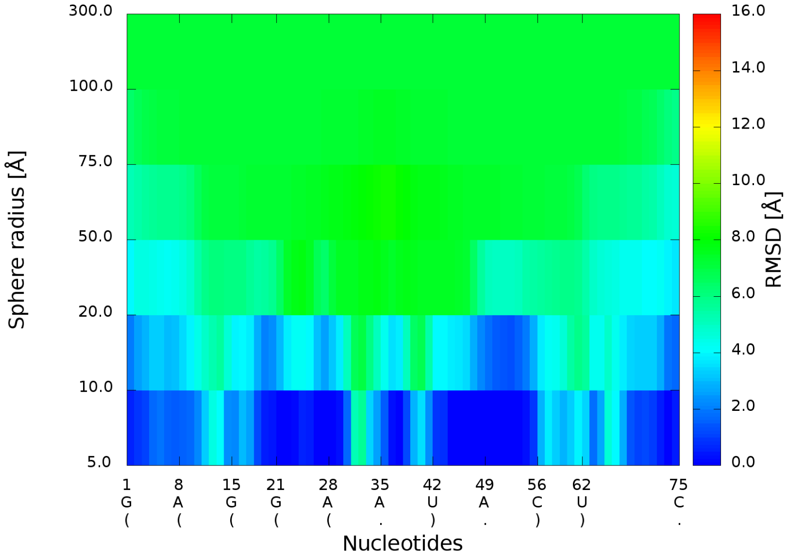

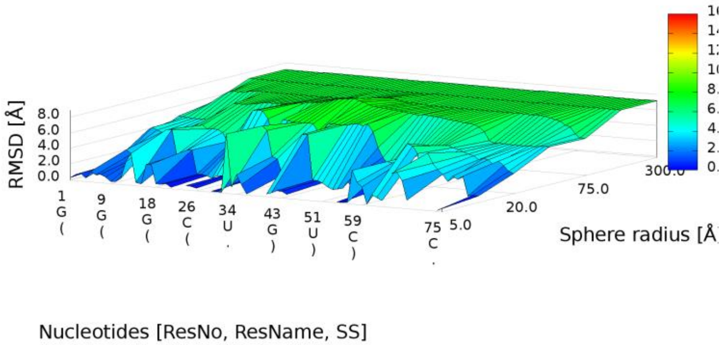

Pseudoscoparipes.pdb

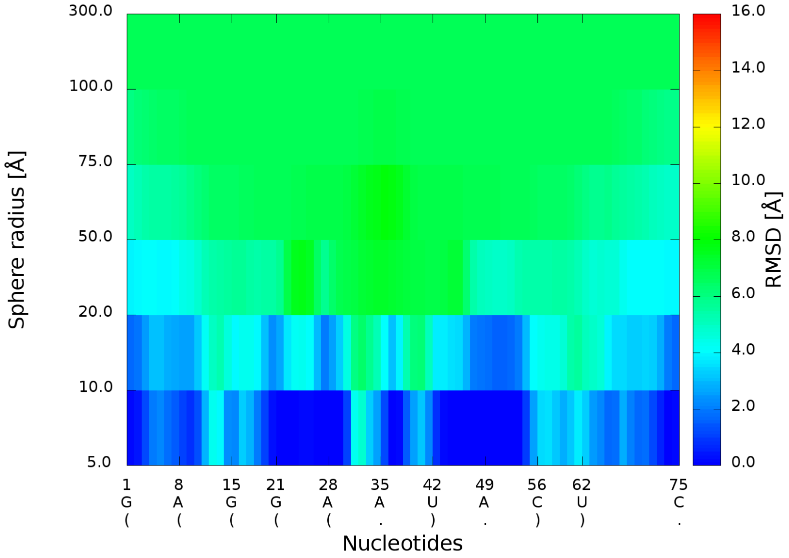

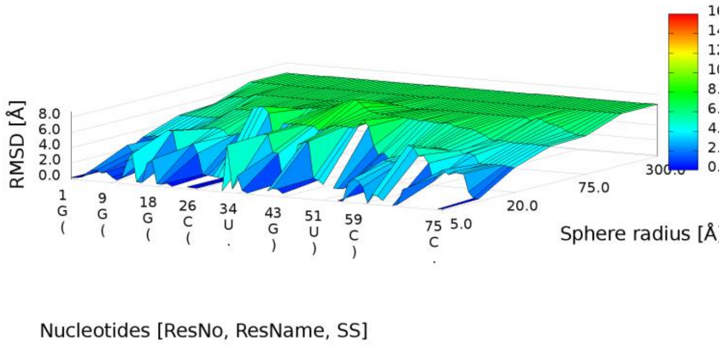

Rhytidoporus.pdb

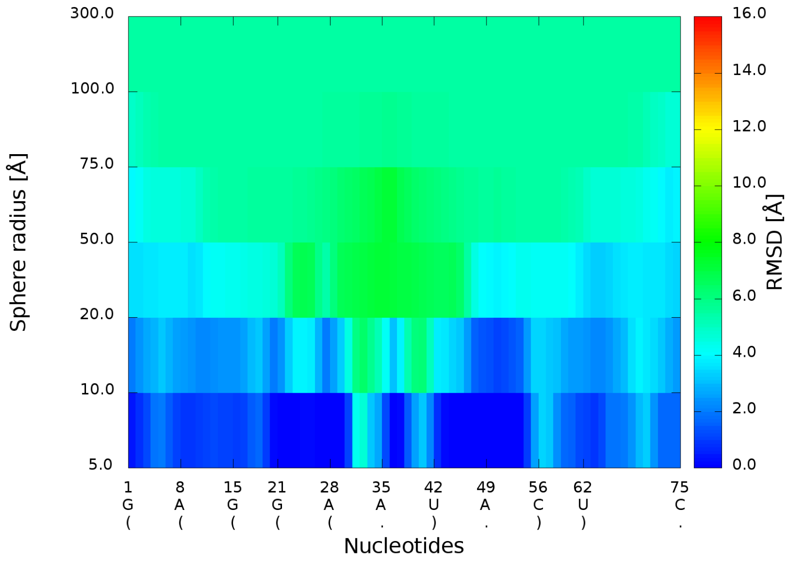

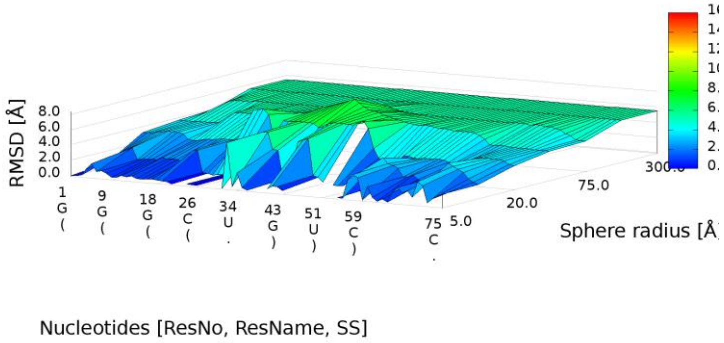

Stibaropus.pdb

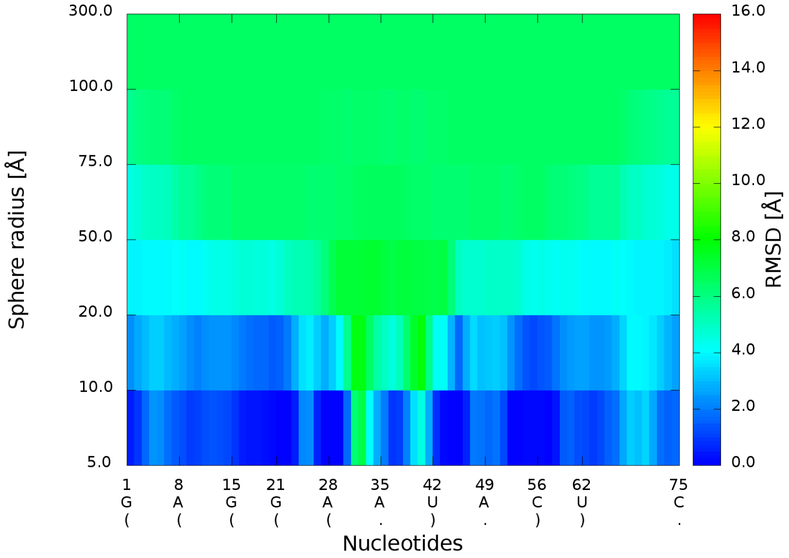

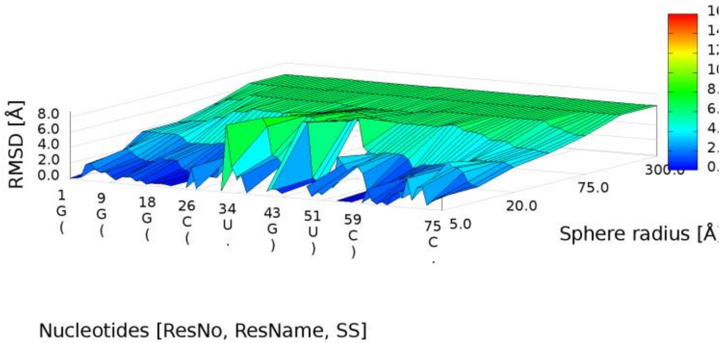

Thyreocoris.pdb

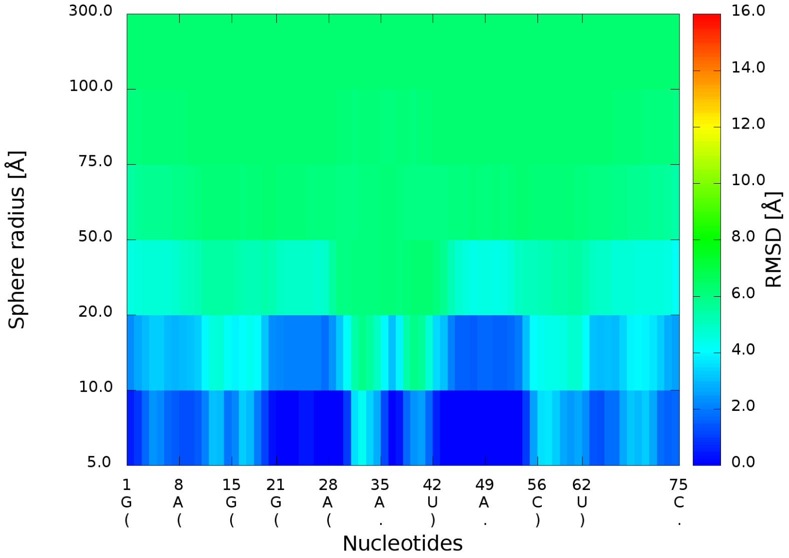

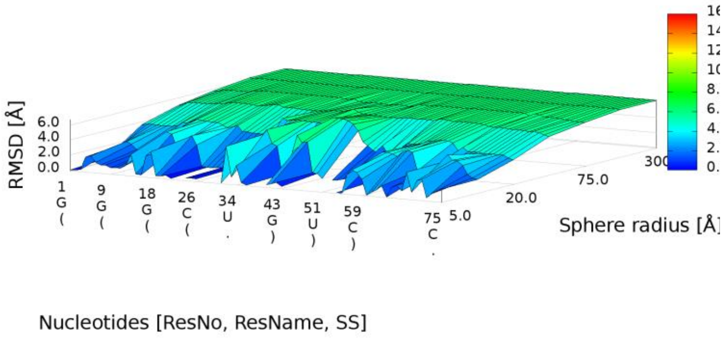

Supplement: Supplementary file 1 [file ijms-25-00939-s001.zip › Supplementary File S3.pdf]
