# Supplementary material for: Inconsistencies in the Classification of the Family Cydnidae (Hemiptera: Heteroptera: Pentatomoidea) Revealed by Molecular Apomorphies in the Secondary and Tertiary Structures of 18S rRNA Length-Variable Region L (LVR L)
Source: Int J Mol Sci. 2024 Jan 11;25(2):939. doi: 10.3390/ijms25020939 (PMC10815949; doi:10.3390/ijms25020939)
Supplement: Supplementary file 1 [file ijms-25-00939-s001.zip › Supplementary Materials.pdf]

Supplementary materials

Figure S1. The secondary and tertiary structure models of the predicted 18S rRNA gene for *Adomerus biguttatus* (subfamily Sehirinae).

Figure S2. Subdividion of the LVR L region into subregions in all analysed sequences.

File S1. The final 18S rDNA hypervariable region V4 alignment (in FASTA format).

File S2. Number of nucleotides in the LVR L subregions of the analysed species, with the assignment to the spermathecal types and facies recovered in the family Cydnidae and classified within the higher taxon (family, subfamily, tribe) of the superfamily Pentatomoidea.

File S3. The results of the detailed analysis of the entire LVR L tertiary structures for the sequences of all analysed ingroup species, including the calculation of the Interaction Network Fidelity (INF), Deformation Profile (DP) and P-value coefficients. Each LVR L sequence were aligned to the outgroup (*Thaumastella elizabethae*, Thaumastellidae) model.

Table S1. The number of nucleotides of the hypervariable region V4, the length-variable region L (LVR L), and the L2 subregion of the LVR L in the *18S rRNA* of the analysed taxa. All analysed species of the Cydnidae were assigned to the particular groups of spermathecal types and facies recovered within the family Cydnidae.

Table S2. The number of nucleotides of the hypervariable region V4, the length-variable region L (LVR L), and the L2 subregion of the LVR L in the *18S rRNA* of the analysed taxa.

Table S3. List of species used for the study with GenBank accession numbers for *18S rDNA* hypervariable region V4.

Table S4. List of specimens used for extraction and amplification during the present study. Their geographic origin, GenBank accession numbers, University of Opole sample numbers, and names of the persons who provided the specimens for analyses are given.
