# Supplementary material for: Inconsistencies in the Classification of the Family Cydnidae (Hemiptera: Heteroptera: Pentatomoidea) Revealed by Molecular Apomorphies in the Secondary and Tertiary Structures of 18S rRNA Length-Variable Region L (LVR L)
Source: Int J Mol Sci. 2024 Jan 11;25(2):939. doi: 10.3390/ijms25020939 (PMC10815949; doi:10.3390/ijms25020939)
Supplement: Supplementary file 1 [file ijms-25-00939-s001.zip › Supplementary Table S1.pdf]

Table S1. The number of nucleotides of the hypervariable region V4, the length-variable region L (LVR L), and the L2 subregion of the LVR L in the *18S rRNA* of the analysed taxa. All analysed species of the Cydnidae were assigned to the particular groups of spermathecal types and facies recovered within the family Cydnidae [19].

**Family**	**Subfamily**	**Spermathecal types / facies** (according to [19])	**Species**	**Number of nucleotides**		
				**V4**	**LVR L**	**L2**
Cydnidae	Amaurocorinae	amaurocorinae type	*Amaurocoris curtus* (Brullé, 1838)	316	73	6
	Amnestinae	amnestinae type	*Amnestus pusio* (Stål, 1860)	324	73	6
			Amnestus zacki Mayorga & Cervantes, 2009	324	73	6
			Lattinestus barrerai Mayorga & Brailovsky, 2012	324	73	6
	Garsauriinae	garsauriinae type	*Garsauria aradoides* Walker, 1868	317	74	6
	Cephalocteinae	"cydnoid" type geotoman facies	*Stibaropus indonesicus* J.A. Lis, 1991	317	74	4
	Cydninae	"cydnoid" type geotoman facies	*Aethus holothrix* Linnavuori, 1977	317	74	4
			Aethus pseudindicus J.A. Lis, 1993	317	74	4
			Alonips obsoletus Signoret, 1881	317	74	4
			Byrsinus pseudosyriacus (Linnavuori, 1977)	317	74	4
			Byrsinus varians (Fabricius, 1803)	317	74	4
			Cyrtomenus emarginatus Stål, 1862	317	74	4
			Fromundus pygmaeus (Dallas, 1851)	317	74	4
			Geotomus convexus Hsiao, 1977	317	74	4
			Lactistes obesipes Signoret, 1879	317	74	4
			Lactistes vericulatus Schiødte, 1848	317	74	4
			Macroscytus badius (Walker, 1867)	317	74	4
			Macroscytus brunneus (Fabricius, 1803)	317	74	4
			Macroscytus fraterculus Horváth, 1919	317	74	4
			Microporus nigrita (Fabricius, 1794)	317	74	4
			Pangaeus bilineatus (Say, 1825)	317	74	4
			Pangaeus rugiceps Horváth, 1919	317	74	4
			Rhytidoporus indentatus Uhler, 1877	317	74	4
		"cydnoid" type adrisan facies	*Adrisa magna* (Uhler, 1861)	316	73	4
			Adrisa romani J.A. Lis, 1994	316	73	4
		"cydnoid" type scoparipan facies	*Pseudoscoparipes fraterculus* J.A. Lis, 1994	316	73	4
			Pseudoscoparipes kinabalensis J.A. Lis, 1994	316	73	4
		"cydnoid" type cydnan facies	*Chilocoris confusus* Horváth, 1919	325	81	7
			Chilocoris laevicollis Horváth, 1919	325	81	7
			Chilocoris piceus Signoret, 1884	325	81	7
			Parachilocoris minutus (Distant, 1901)	325	81	7
			Cydnus aterrimus (Forster, 1771)	316	73	4
	Sehirinae	"cydnoid type" sehiran facies	*Adomerus biguttatus* (Linnaeus, 1758)	316	73	6
			Canthophorus niveimarginatus Scott, 1874	316	73	6
			Sehirus luctuosus Mulsant et Rey, 1866	316	73	6

			Tritomegas bicolor (Linnaeus, 1758)	316	73	6
			Tritomegas sexmaculatus (Rambur, 1839)	316	73	6
		"cydnoid type" ochetostethan facies	*Ochetostethomorpha secunda* J.A. Lis & B. Lis, 2014	316	73	4
			Ochetostethus opacus (Scholtz, 1847)	316	73	4
Parastrachiidae	–	–	*Dismegistus sanguineus* (DeGeer, 1778)	316	73	6
			Parastrachia japonensis (Scott, 1880)	316	73	6
Thyreocoridae	Corimelaeninae	–	*Galgupha australis* McAtee & Malloch, 1933	316	74	6
	Thyreocorinae	–	*Thyreocoris scarabaeoides* (Linnaeus, 1758)	316	74	6
Thaumastellidae (outgroup)	–	–	*Thaumastella elizabethae* Jacobs, 1989	318	75	3
			Thaumastella namaquensis Schaefer & Wilcox, 1971	315	72	3
