# Supplementary material for: Inconsistencies in the Classification of the Family Cydnidae (Hemiptera: Heteroptera: Pentatomoidea) Revealed by Molecular Apomorphies in the Secondary and Tertiary Structures of 18S rRNA Length-Variable Region L (LVR L)
Source: Int J Mol Sci. 2024 Jan 11;25(2):939. doi: 10.3390/ijms25020939 (PMC10815949; doi:10.3390/ijms25020939)
Supplement: Supplementary file 1 [file ijms-25-00939-s001.zip › Supplementary Table S2.pdf]

Table S2. The number of nucleotides of the hypervariable region V4, the length-variable region L (LVR L), and the L2 subregion of the LVR L in the *18S rRNA* of the analysed taxa.

**Region**	**Number of nucleotides**	**Species**	**Subfamily**	**Family**
**V4**	**315**	*Thaumastella namaquensis* Schaefer & Wilcox, 1971	–	Thaumastellidae
	**316**	*Amaurocoris curtus* (Brullé, 1838)	Amaurocorinae	Cydnidae
		Adrisa magna (Uhler, 1861)	Cydninae	
		Adrisa romani J.A. Lis, 1994		
		Pseudoscoparipes fraterculus J.A. Lis, 1994		
		Pseudoscoparipes kinabalensis J.A. Lis, 1994		
		Cydnus aterrimus (Forster, 1771)		
		Adomerus biguttatus (Linnaeus, 1758)	Sehirinae	
		Canthophorus niveimarginatus Scott, 1874		
		Sehirus luctuosus Mulsant et Rey, 1866		
		Tritomegas bicolor (Linnaeus, 1758)		
		Tritomegas sexmaculatus (Rambur, 1839)		
		Ochetostethomorpha secunda J.A. Lis & B. Lis, 2014		
		Ochetostethus opacus (Scholtz, 1847)		
		Dismegistus sanguineus (DeGeer, 1778)	–	Parastrachiidae
		Parastrachia japonensis (Scott, 1880)		
		Galgupha australis McAtee & Malloch, 1933	Corimelaeninae	Thyreocoridae
		Thyreocoris scarabaeoides (Linnaeus, 1758)	Thyreocorinae	
	**317**	*Garsauria aradoides* Walker, 1868	Garsauriinae	Cydnidae
		Stibaropus indonesicus J.A. Lis, 1991	Cephalocteinae	
		Aethus holothrix Linnavuori, 1977	Cydninae	
		Aethus pseudindicus J.A. Lis, 1993		
		Alonips obsoletus Signoret, 1881		
		Byrsinus pseudosyriacus (Linnavuori, 1977)		
		Byrsinus varians (Fabricius, 1803)		
		Cyrtomenus emarginatus Stål, 1862		
		Fromundus pygmaeus (Dallas, 1851)		
		Geotomus convexus Hsiao, 1977		
		Lactistes obesipes Signoret, 1879		
		Lactistes vericulatus Schiødte, 1848		

	**317**	*Macroscytus badius* (Walker, 1867)	Cydninae	Cydnidae
		Macroscytus brunneus (Fabricius, 1803)		
		Macroscytus fraterculus Horváth, 1919		
		Microporus nigrita (Fabricius, 1794)		
		Pangaeus bilineatus (Say, 1825)		
		Pangaeus rugiceps Horváth, 1919		
		Rhytidoporus indentatus Uhler, 1877		
	**318**	*Thaumastella elizabethae* Jacobs, 1989	–	Thaumastellidae
	**324**	*Amnestus pusio* (Stål, 1860)	Amnestinae	Cydnidae
		Amnestus zacki Mayorga & Cervantes, 2009		
		Lattinestus barrerai Mayorga & Brailovsky, 2012		
	**325**	*Chilocoris confusus* Horváth, 1919	Cydninae	Cydnidae
		Chilocoris laevicollis Horváth, 1919		
		Chilocoris piceus Signoret, 1884		
		Parachilocoris minutus (Distant, 1901)		
**LVR L**	**72**	*Thaumastella namaquensis* Schaefer & Wilcox, 1971		Thaumastellidae
	**73**	*Amaurocoris curtus* (Brullé, 1838)	Amaurocorinae	Cydnidae
		Amnestus pusio (Stål, 1860)	Amnestinae	
		Amnestus zacki Mayorga & Cervantes, 2009		
		Lattinestus barrerai Mayorga & Brailovsky, 2012		
		Adrisa magna (Uhler, 1861)	Cydninae	
		Adrisa romani J.A. Lis, 1994		
		Pseudoscoparipes fraterculus J.A. Lis, 1994		
		Pseudoscoparipes kinabalensis J.A. Lis, 1994		
		Cydnus aterrimus (Forster, 1771)		
		Adomerus biguttatus (Linnaeus, 1758)	Sehirinae	
		Canthophorus niveimarginatus Scott, 1874		
		Sehirus luctuosus Mulsant et Rey, 1866		
		Tritomegas bicolor (Linnaeus, 1758)		
		Tritomegas sexmaculatus (Rambur, 1839)		
		Ochetostethomorpha secunda J.A. Lis & B. Lis, 2014		
		Ochetostethus opacus (Scholtz, 1847)		
		Dismegistus sanguineus (DeGeer, 1778)	–	Parastrachiidae
		Parastrachia japonensis (Scott, 1880)		
	**74**	*Garsauria aradoides* Walker, 1868	Garsauriinae	Cydnidae

	**74**	*Stibaropus indonesicus* J.A. Lis, 1991	Cephalocteinae	Cydnidae
		Aethus holothrix Linnavuori, 1977	Cydninae	
		Aethus pseudindicus J.A. Lis, 1993		
		Alonips obsoletus Signoret, 1881		
		Byrsinus pseudosyriacus (Linnavuori, 1977)		
		Byrsinus varians (Fabricius, 1803)		
		Cyrtomenus emarginatus Stål, 1862		
		Fromundus pygmaeus (Dallas, 1851)		
		Geotomus convexus Hsiao, 1977		
		Lactistes obesipes Signoret, 1879		
		Lactistes vericulatus Schiødte, 1848		
		Macroscytus badius (Walker, 1867)		
		Macroscytus brunneus (Fabricius, 1803)		
		Macroscytus fraterculus Horváth, 1919		
		Microporus nigrita (Fabricius, 1794)		
		Pangaeus bilineatus (Say, 1825)		
		Pangaeus rugiceps Horváth, 1919		
		Rhytidoporus indentatus Uhler, 1877		
		Galgupha australis McAtee & Malloch, 1933	Corimelaeninae	Thyreocoridae
		Thyreocoris scarabaeoides (Linnaeus, 1758)	Thyreocorinae	
	**75**	*Thaumastella elizabethae* Jacobs, 1989	–	Thaumastellidae
	**81**	*Chilocoris confusus* Horváth, 1919	Cydninae	Cydnidae
		Chilocoris laevicollis Horváth, 1919		
		Chilocoris piceus Signoret, 1884		
		Parachilocoris minutus (Distant, 1901)		
**L2**	**3**	*Thaumastella elizabethae* Jacobs, 1989	–	Thaumastellidae
		Thaumastella namaquensis Schaefer & Wilcox, 1971		
	**4**	*Stibaropus indonesicus* J.A. Lis, 1991	Cephalocteinae	Cydnidae
		Aethus holothrix Linnavuori, 1977	Cydninae	
		Aethus pseudindicus J.A. Lis, 1993		
		Alonips obsoletus Signoret, 1881		
		Byrsinus pseudosyriacus (Linnavuori, 1977)		
		Byrsinus varians (Fabricius, 1803)		
		Cyrtomenus emarginatus Stål, 1862		
		Fromundus pygmaeus (Dallas, 1851)		

**4**	*Geotomus convexus* Hsiao, 1977	Cydninae	Cydnidae
	Lactistes obesipes Signoret, 1879		
	Lactistes vericulatus Schiødte, 1848		
	Macroscytus badius (Walker, 1867)		
	Macroscytus brunneus (Fabricius, 1803)		
	Macroscytus fraterculus Horváth, 1919		
	Microporus nigrita (Fabricius, 1794)		
	Pangaeus bilineatus (Say, 1825)		
	Pangaeus rugiceps Horváth, 1919		
	Rhytidoporus indentatus Uhler, 1877		
	Adrisa magna (Uhler, 1861)		
	Adrisa romani J.A. Lis, 1994		
	Pseudoscoparipes fraterculus J.A. Lis, 1994		
	Pseudoscoparipes kinabalensis J.A. Lis, 1994		
	Cydnus aterrimus (Forster, 1771)		
	Ochetostethomorpha secunda J.A. Lis & B. Lis, 2014	Sehirinae	
	Ochetostethus opacus (Scholtz, 1847)		
**6**	*Amaurocoris curtus* (Brullé, 1838)	Amaurocorinae	Cydnidae
	Garsauria aradoides Walker, 1868	Garsauriinae	
	Amnestus pusio (Stål, 1860)	Amnestinae	
	Amnestus zacki Mayorga & Cervantes, 2009		
	Lattinestus barrerai Mayorga & Brailovsky, 2012		
	Adomerus biguttatus (Linnaeus, 1758)	Sehirinae	
	Canthophorus niveimarginatus Scott, 1874		
	Sehirus luctuosus Mulsant et Rey, 1866		
	Tritomegas bicolor (Linnaeus, 1758)		
	Tritomegas sexmaculatus (Rambur, 1839)		
	Dismegistus sanguineus (DeGeer, 1778)	–	Parastrachiidae
	Parastrachia japonensis (Scott, 1880)		
	Galgupha australis McAtee & Malloch, 1933	Corimelaeninae	Thyreocoridae
	Thyreocoris scarabaeoides (Linnaeus, 1758)	Thyreocorinae	
**7**	*Chilocoris confusus* Horváth, 1919	Cydninae	Cydnidae
	Chilocoris laevicollis Horváth, 1919		
	Chilocoris piceus Signoret, 1884		
	Parachilocoris minutus (Distant, 1901)		
