# Supplementary material for: Inconsistencies in the Classification of the Family Cydnidae (Hemiptera: Heteroptera: Pentatomoidea) Revealed by Molecular Apomorphies in the Secondary and Tertiary Structures of 18S rRNA Length-Variable Region L (LVR L)
Source: Int J Mol Sci. 2024 Jan 11;25(2):939. doi: 10.3390/ijms25020939 (PMC10815949; doi:10.3390/ijms25020939)
Supplement: Supplementary file 1 [file ijms-25-00939-s001.zip › Supplementary Table S3.pdf]

Table S3. List of species used for the study with GenBank accession numbers for *18S rDNA* hypervariable region V4.

Family	Subfamily	Species	Geographic origin	GenBank accesion number for the 18S rDNA hypervariable region V4	Source
Cydnidae	Amaurocorinae	*Amaurocoris curtus* (Brullé, 1838)	Cyprus	OR691635	present study
	Amnestinae	*Amnestus pusio* (Stål, 1860)	Guatemala	OR691636	present study
		Amnestus zacki Mayorga & Cervantes, 2009	Guatemala	OR691637	present study
		Lattinestus barrerai Mayorga & Brailovsky, 2012	Guatemala	OR691650	present study
	Cephalocteinae	*Stibaropus indonesicus* J.A. Lis, 1991	Brunei	OR691659	present study
	Garsauriinae	*Garsauria aradoides* Walker, 1868	Brunei	OR691647	present study
	Cydninae	*Adrisa magna* (Uhler, 1861)	Japan	KY911199	[1]
		Adrisa romani J.A. Lis, 1994	Thailand	OR691631	present study
		Aethus holothrix Linnavuori, 1977	Namibia	OR691632	present study
		Aethus pseudindicus J.A. Lis, 1993	Vietnam	OR691633	present study
		Alonips obsoletus Signoret, 1881	Australia	OR691634	present study
		Byrsinus pseudosyriacus (Linnavuori, 1977)	Namibia	OR691638	present study
		Byrsinus varians (Fabricius, 1803)	Guam	OR691639	present study
		Chilocoris confusus Horváth, 1919	Japan	OR691641	present study
		Chilocoris laevicollis Horváth, 1919	Kenya	OR691642	present study
		Chilocoris piceus Signoret, 1884	Thailand	OR691643	present study
		Cydnus aterrimus (Forster, 1771)	Italy	KY911202	[1]
		Cyrtomenus emarginatus Stål, 1862	Guatemala	OR691644	present study
		Fromundus pygmaeus (Dallas, 1851)	Taiwan	KJ535871	[2]
		Geotomus convexus Hsiao, 1977	Japan	KY911203	[1]
		Lactistes obesipes Signoret, 1879	Australia	OR691648	present study
		Lactistes vericulatus Schiødte, 1848	Zambia	OR691649	present study
		Macroscytus badius (Walker, 1867)	India	OR691651	present study
		Macroscytus brunneus (Fabricius, 1803)	Namibia	KY911204	[1]
		Macroscytus fraterculus Horváth, 1919	Japan	OR691652	present study
		Microporus nigrita (Fabricius, 1794)	Poland	KY911205	[1]
		Pangaeus bilineatus (Say, 1825)	Guatemala	KY911207	[1]
		Pangaeus rugiceps Horváth, 1919	Guatemala	OR691654	present study
		Parachilocoris minutus (Distant, 1901)	Japan	OR691655	present study

		Pseudoscoparipes fraterculus J.A. Lis, 1994	Thailand	OR691656	present study
		Pseudoscoparipes kinabalensis J.A. Lis, 1994	Brunei	OR691657	present study
		Rhytidoporus indentatus Uhler, 1877	Guam	KY911208	[1]
	Sehirinae	*Adomerus biguttatus* (Linnaeus, 1758)	Poland	KY911198	[1]
		Canthophorus niveimarginatus Scott, 1874	Japan	OR691640	present study
		Ochetostethomorpha secunda J.A. Lis & B. Lis, 2014	Namibia	KY911206	[1]
		Ochetostethus opacus (Scholtz, 1847)	Poland	OR691653	present study
		Sehirus luctuosus Mulsant et Rey, 1866	Poland	OR691658	present study
		Tritomegas bicolor (Linnaeus, 1758)	Poland	OR691660	present study
		Tritomegas sexmaculatus (Rambur, 1839)	Poland	KY911210	[1]
Parastrachiidae		*Dismegistus sanguineus* (DeGeer, 1778)	Namibia (2012)	OR691645	present study
		Parastrachia japonensis (Scott, 1880)	Japan	EF641204	[3]
Thyreocoridae	Corimelaeninae	*Galgupha australis* McAtee & Malloch, 1933	Bolivia	OR691646	present study
	Thyreocorinae	*Thyreocoris scarabaeoides* (Linnaeus, 1758)	Poland	KY911214	[1]
Thaumastellidae (outgroup)		*Thaumastella elizabethae* Jacobs, 1989	South Africa	EF641221	[3]
		Thaumastella namaquensis Schaefer & Wilcox, 1971	South Africa	EF641222	[3]

References

1. Lis J.A., Ziaja D., Lis B. & Gradowska P.A. 2017. Non-monophyly of the "cydnoid" complex within Pentatomoidea (Hemiptera: Heteroptera) revealed by Bayesian phylogenetic analysis of nuclear rDNA sequences. *Arthropod Systematics & Phylogeny*, **75**, 481–496.
2. Wu Y-Z., Yu S-S., Wang Y-H., Wu H-Y., Li X-R., Men X-Y., Zhang Y-W., Rédei D., Xie Q., Bu W-J. 2016. The evolutionary position of Lestoniidae revealed by molecular autapomorphies in the secondary structure of rRNA besides phylogenetic reconstruction (Insecta: Hemiptera: Heteroptera). *Zoological Journal of the Linnean Society* **1771**: 750–763.
3. Grazia J., Schuh R.T., Wheeler W.C. 2008. Phylogenetic relationships of family groups in Pentatomoidea based on morphology and DNA sequences (Insecta: Heteroptera). *Cladistics* **24**: 1–45.
