# Supplementary material for: Inconsistencies in the Classification of the Family Cydnidae (Hemiptera: Heteroptera: Pentatomoidea) Revealed by Molecular Apomorphies in the Secondary and Tertiary Structures of 18S rRNA Length-Variable Region L (LVR L)
Source: Int J Mol Sci. 2024 Jan 11;25(2):939. doi: 10.3390/ijms25020939 (PMC10815949; doi:10.3390/ijms25020939)
Supplement: Supplementary file 1 [file ijms-25-00939-s001.zip › Supplementary Table S4.pdf]

Table S4. List of specimens used for extraction and amplification during the present study. Their geographic origin, GenBank accession numbers, University of Opole sample numbers, and names of the persons who provided the specimens for analyses are given. All newly sequenced specimens were identified to species by the first author (JAL). Acronyms for the persons who collected or/and provided the specimens for analyses are, as follows: AT (Artur Taszakowski, University of Silesia in Katowice, Faculty of Natural Sciences, Institute of Biology, Biotechnology and Environmental Protection, Katowice, Poland), AW (Andrzej Wolski, Institute of Biology, University of Opole, Opole, Poland), BL (Barbara Lis, Institute of Biology, University of Opole, Opole, Poland), CD (Claas Damken, Stadland, Germany), HB (Harry Brailovsky, Instituto de Biología, Universidad Nacional Autónoma de México, D.F., México), HVG (Hemant V. Ghate, Modern College of Arts Science and Commerce, Shivajinagar, Pune, Maharashtra, India), LB (Luca Bartolozzi, Department of Entomology, Natural History Museum of the University of Florence, Zoological Section "La Specola", Florence, Italy), LC (Lawrence Cockson, Warrandyte, Victoria, Australia), PJD (Paweł J. Domagała, Institute of Biology, University of Opole, Opole, Poland), RD (Roland Dobosz, Department of Natural History, Upper Silesian Museum, Bytom, Poland),RSZ (Richard S. Zack, Department of Entomology, Washington State University, Pullman, Washington, U.S.A.), SK (Shin-ichi Kudo, Department of Biology, Naruto University of Education, Naruto,Tokushima, Japan).

Family	Subfamily	Tribe	Species/subspecies	Geographic origin (year collected, if originally provided)	GenBank accesion numbers for the 18S rDNA hypervariable region V4	University of Opole sample numbers for newly sequenced species (person who provided or/and collected the specimen)
Cydnidae	Amaurocorinae	-	*Amaurocoris curtus* (Brullé, 1838)	Cyprus (2015)	OR691635	UO_226 (AT)
	Amnestinae	-	*Amnestus pusio* (Stål, 1860)	Guatemala (2011)	OR691636	UO_153Ap (RSZ)
			Amnestus zacki Mayorga & Cervantes, 2009	Guatemala (2010)	OR691637	UO_134Az (RSZ)
			Lattinestus barrerai Mayorga & Brailovsky, 2012	Guatemala (2007)	OR691650	UO_233Lb (HB)
	Cephalocteinae	Scaptocorini	*Stibaropus indonesicus* J.A. Lis, 1991	Brunei (2014)	OR691659	UO_218Ssp (CD)
	Garsauriinae	-	*Garsauria aradoides* Walker, 1868	Brunei (2014)	OR691647	UO_217Ga (CD)
	Cydninae	Cydnini	*Chilocoris confusus* Horváth, 1919	Japan (2011)	OR691641	UO_166 (SK)
			Chilocoris laevicollis Horváth, 1919	Kenya (2013)	OR691642	UO_3Cl (LB)
			Chilocoris piceus Signoret, 1884	Thailand (2013)	OR691643	UO_8Cp (AW)
			Parachilocoris minutus (Distant, 1901)	Japan (2011)	OR691655	UO_168 (SK)
		Geotomini	*Alonips obsoletus* Signoret, 1881	Australia (2018)	OR691634	UO_13Ao (LC)
			Aethus holothrix Linnavuori, 1977	Namibia (2012)	OR691632	UO_24Ah (RD)
			Aethus pseudindicus J.A. Lis, 1993	Vietnam	OR691633	UO_7Ap (LC)
			Adrisa romani J.A. Lis, 1994	Thailand (2013)	OR691631	UO_10Ar (AW)
			Byrsinus pseudosyriacus (Linnavuori, 1977)	Namibia (2012)	OR691638	UO_22Bp (RD)
			Byrsinus varians (Fabricius, 1803)	Guam (2004)	OR691639	UO_149Bv (RSZ)
			Cyrtomenus emarginatus Stål, 1862	Guatemala (2011)	OR691644	UO_152Ce (RSZ)
			Lactistes obesipes Signoret, 1879	Australia (2018)	OR691648	UO_14Lo (LC)

			Lactistes vericulatus Schiødte, 1848	Zambia (2014)	OR691649	UO_15Lv (PJD)
			Macroscytus badius (Walker, 1867)	India (2013)	OR691651	UO_1Mb (HVG)
			Macroscytus fraterculus Horváth, 1919	Japan (2010)	OR691652	UO_142 (SK)
			Pangaeus rugiceps Horváth, 1919	Guatemala (2011)	OR691654	UO_171Pr (RSZ)
			Pseudoscoparipes fraterculus J.A. Lis, 1994	Thailand (2013)	OR691656	UO_20Pb (AW)
			Pseudoscoparipes kinabalensis J.A. Lis, 1994	Brunei (2015)	OR691657	UO_219Pk (CD)
	Sehirinae	Sehirini	*Canthophorus niveimarginatus* Scott, 1874	Japan (2010)	OR691640	UO_141 (SK)
			Ochetostethus opacus (Scholtz, 1847)	Poland (2016)	OR691653	UO_223Oo (AT)
			Sehirus luctuosus Mulsant et Rey, 1866	Poland (2014)	OR691658	UO_186Sl (BL)
			Tritomegas bicolor (Linnaeus, 1758)	Poland (2011)	OR691660	UO_144 (BL)
Parastrachiidae	-	-	*Dismegistus sanguineus* (De Geer, 1778)	Namibia (2012)	OR691645	UO_38 (RD)
Thyreocoridae	Corimelaeninae	-	*Galgupha australis* McAtee & Malloch, 1933	Bolivia (2010)	OR691646	UO_163A (AW)
