# Supplementary figures and images for: Inconsistencies in the Classification of the Family Cydnidae (Hemiptera: Heteroptera: Pentatomoidea) Revealed by Molecular Apomorphies in the Secondary and Tertiary Structures of 18S rRNA Length-Variable Region L (LVR L)
Source: Int J Mol Sci. 2024 Jan 11;25(2):939. doi: 10.3390/ijms25020939 (PMC10815949; doi:10.3390/ijms25020939)

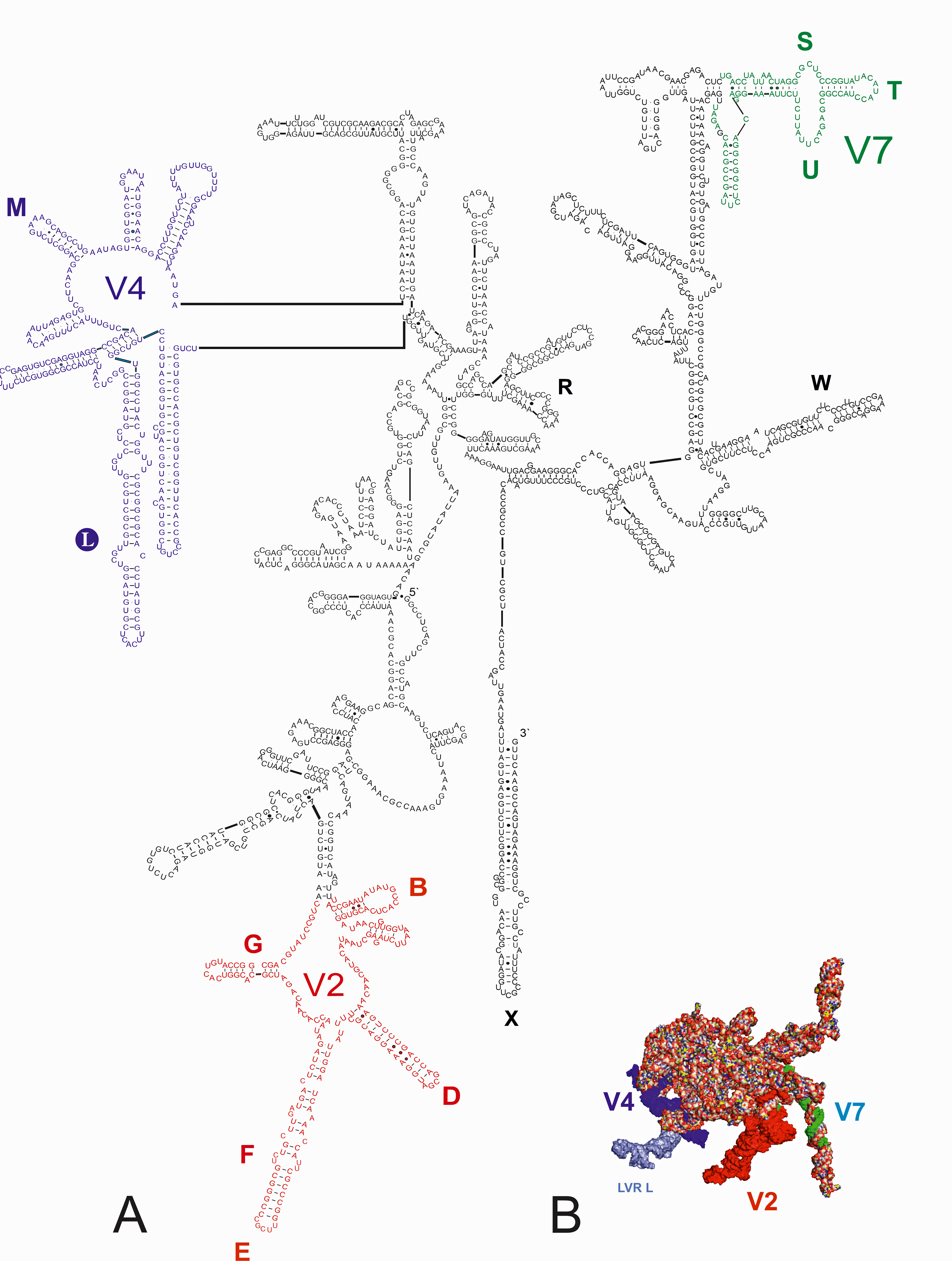

Supplement: Supplementary file 1 [file ijms-25-00939-s001.zip › Supplementary Figure S1.tif]

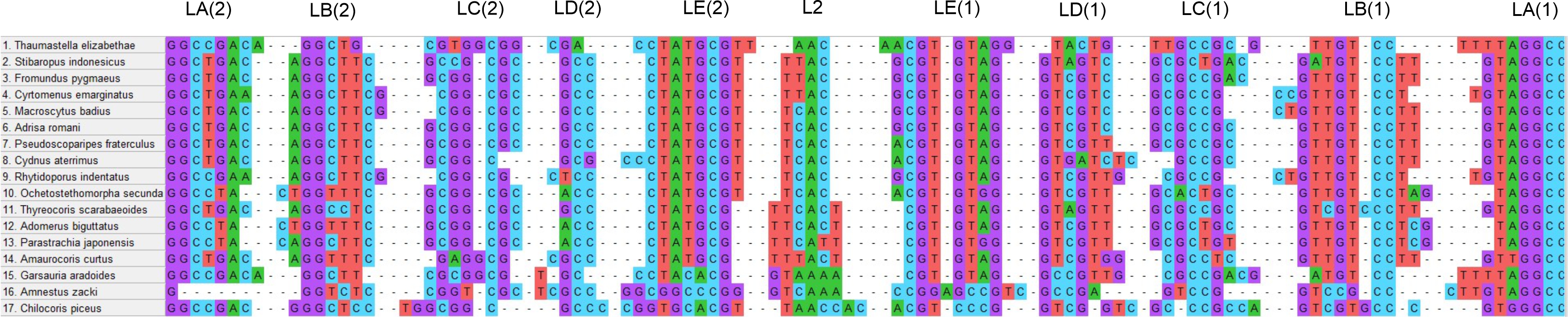

Supplement: Supplementary file 1 [file ijms-25-00939-s001.zip › Supplementary Figure S2.jpg]
